# Randomized boosting with multivariable base-learners for high-dimensional variable selection and prediction

**DOI:** 10.1186/s12859-021-04340-z

**Published:** 2021-09-16

**Authors:** Christian Staerk, Andreas Mayr

**Affiliations:** Department of Medical Biometry, Informatics and Epidemiology, University Hospital Bonn, Venusberg-Campus 1, 53127 Bonn, Germany

**Keywords:** Boosting, Feature selection, High-dimensional data, Information criteria, Sparsity, Variable selection

## Abstract

**Background:**

Statistical boosting is a computational approach to select and estimate interpretable prediction models for high-dimensional biomedical data, leading to implicit regularization and variable selection when combined with early stopping. Traditionally, the set of base-learners is fixed for all iterations and consists of simple regression learners including only one predictor variable at a time. Furthermore, the number of iterations is typically tuned by optimizing the predictive performance, leading to models which often include unnecessarily large numbers of noise variables.

**Results:**

We propose three consecutive extensions of classical component-wise gradient boosting. In the first extension, called Subspace Boosting (SubBoost), base-learners can consist of several variables, allowing for multivariable updates in a single iteration. To compensate for the larger flexibility, the ultimate selection of base-learners is based on information criteria leading to an automatic stopping of the algorithm. As the second extension, Random Subspace Boosting (RSubBoost) additionally includes a random preselection of base-learners in each iteration, enabling the scalability to high-dimensional data. In a third extension, called Adaptive Subspace Boosting (AdaSubBoost), an adaptive random preselection of base-learners is considered, focusing on base-learners which have proven to be predictive in previous iterations. Simulation results show that the multivariable updates in the three subspace algorithms are particularly beneficial in cases of high correlations among signal covariates. In several biomedical applications the proposed algorithms tend to yield sparser models than classical statistical boosting, while showing a very competitive predictive performance also compared to penalized regression approaches like the (relaxed) lasso and the elastic net.

**Conclusions:**

The proposed randomized boosting approaches with multivariable base-learners are promising extensions of statistical boosting, particularly suited for highly-correlated and sparse high-dimensional settings. The incorporated selection of base-learners via information criteria induces automatic stopping of the algorithms, promoting sparser and more interpretable prediction models.

**Supplementary information:**

The online version contains supplementary material available at 10.1186/s12859-021-04340-z.

## Background

The increasing availability of high-dimensional biomedical data with many possible predictor variables calls for appropriate statistical tools in order to deal with the challenging problem of selecting an interpretable model that includes only the relevant variables for modelling a particular outcome. At the same time, it is desirable that the prediction accuracy is not deteriorated by selecting an overly sparse model.

Various variable selection methods have been proposed in the context of high-dimensional regression (see Table 1). Regularization approaches minimize the empirical risk function while considering additional penalties on the “size” of the regression coefficients, including the lasso [1] and the relaxed lasso [2, 3] with an $\ell _1$-penalty as well as the the elastic net [4] with a combined $\ell _1$- and $\ell _2$-penalty. These methods yield sparse point estimates through the imposed penalties, which enforce shrinkage of the regression coefficients towards zero; particularly, several coefficients are estimated to be exactly zero, corresponding to the exclusion of the respective variables from the model. A viable alternative to regularization methods is statistical boosting (see e.g. [5–7]). The general concept is best illustrated with the squared error loss, for which two important variants of statistical boosting—gradient boosting [8] and likelihood-based boosting [9]—yield basically the same algorithm called $L_2$Boosting [10, 11]. In each iteration of $L_2$Boosting the currently estimated regression coefficient vector is updated by adding one of several prespecified base-learners that leads to the best fit of the current residuals (i.e. of the negative gradient of the empirical risk function). The base-learners are typically defined by simple regression models each including one of the covariates (known as component-wise boosting) and the starting point is chosen as the zero regression vector, so that early stopping of the boosting algorithm leads to implicit regularization and variable selection.

It has been shown that there is a close connection between the lasso and $L_2$Boosting [12–14] and that the performance of both methods is often very similar in practice [15]. However, an important difference is that the lasso enforces regularization explicitly via the definition of the $\ell _1$-penalized optimization problem, whereas the regularization in boosting is imposed rather indirectly via early stopping of the algorithm after a finite number of iterations. While the explicit form of regularization in methods like the lasso can facilitate the theoretical analysis of the resulting estimators (see e.g. [16]), the implicit algorithmic regularization of boosting offers a large flexibility regarding the choice of the base-learners, enabling the application of boosting on a variety of different models, which can include non-linear covariate effects as in generalized additive models (GAMs) [9] or in generalized additive models for location, scale, and shape (GAMLSS) [17].

In practice, the choice of the penalty parameter in the lasso and the choice of the number of iterations in boosting are crucial, since they control the amount of imposed regularization and sparsity. The tuning of these parameters is typically guided by optimizing the predictive performance (e.g. via cross-validation), leading to final models which often include unnecessarily large numbers of noise variables with small effects. Stability selection [18–21] is a resampling technique that aims to reduce and control the number of selected false positives by applying a variable selection method on several subsamples of the observed data. However, the strict control of false positives by stability selection can induce a considerable reduction of selected variables which are truly relevant for modelling the response, leading to sparse models with poor predictive performance (cf. [22]).

By construction, boosting methods are “greedy” similar to forward stagewise algorithms: once a coefficient is updated at some point of the regularization path, the corresponding variable will be included in all more complex models along the path, although the contribution to the outcome may be small. Further, it has been shown that noise variables tend to be selected early on the lasso regularization path, even in favorable situations with low correlations between the covariates [23]. Thus, the regularization paths induced by classical boosting and the lasso are often too restrictive in order to simultaneously achieve a small false positive rate (sparsity) and a small false negative rate with good predictive performance.

**Table 1:** Selective summary of variable selection methods with types of regularizers, main regularization parameters and computational efficiency. Here we focus on the main regularization parameters of the different methods, but there are often several additional hyper-parameters

Method	Regularizer (parameters)	Comments on computational efficiency
*Explicit regularization*		
Information criteria (4), e.g. AIC [24], BIC [25], EBIC [26]	$\ell _0$-penalty ($\lambda$)	Best subset selection not efficient for high-dimensional problems. Heuristic optimization [27–29] or mixed-integer optimization [30] can be used.
Lasso [1]	$\ell _1$-penalty ($\lambda$)	Computationally efficient convex relaxation of $\ell _0$-type problem.
Relaxed lasso [2, 3]	$\ell _1$-penalty ($\lambda , \gamma$)	Combination of $\ell _1$-regularized and unregularized (restricted least squares) estimator. Computationally efficient but tuning more costly than for lasso.
Elastic net [4]	$\ell_1$-/$\ell_2$-penalty ($\lambda , \alpha$)	Combination of $\ell _1$- and $\ell _2$-penalties. Computationally efficient but tuning more costly than for lasso.
*Implicit regularization*		
$L_2$Boosting [10] (Algorithm 1)	Early stopping ($m_{{\text {stop}}}$)	Tuning of stopping iteration $m_{{\text {stop}}}$ via resampling leads to implicit regularization.
Twin boosting [31]	Early stopping ($m_1,m_2$)	Two-stage approach using $L_2$Boosting estimates as weights in second stage of $L_2$Boosting. Tuning more costly than for single-stage $L_2$Boosting.
Stability selection [18–20]	Flexible (PFER)	Computationally intensive ensemble approach, applying e.g. lasso or $L_2$Boosting multiple times on subsamples. Provides control over false positives (PFER).
New Subspace Boosting: SubBoost (Algorithm 2), RSubBoost and AdaSubBoost (Algorithm 3)	Automatic stopping ($\Phi$)	Multivariable base-learners with *double checking* via selection criterion $\Phi$ for automatic stopping. Randomized preselection of base-learners for scalability. For further hyper-parameters see Algorithms 2 and 3 and the Additional file 1 for their effects on the computational efficiency.

In this work we further exploit the algorithmic flexibility of boosting to address these issues. Here, the primary aim is not the application of boosting to more complex models; instead we reconsider the classical $L_2$Boosting algorithm in the context of high-dimensional linear regression and propose three consecutive extensions of the algorithm with regard to the choice of base-learners, aiming for more flexible regularization paths and sparser final estimators. Traditionally, the set of possible base-learners is fixed for all iterations of boosting and consists of simple regression models including only one covariate at a time. However, this choice is not imperative and may not be optimal: if for example two covariates are highly correlated, then it can be beneficial to update the corresponding regression coefficients jointly in one boosting iteration rather than separately in distinct iterations [32].

In our first extension, called Subspace Boosting (SubBoost), base-learners can consist of several variables so that multiple coefficients may be updated at a single iteration of the algorithm. In order to compensate for the larger flexibility in the choice of the base-learners and to avoid overfitting, in each iteration the final selection is based on likelihood-based $\ell _0$-type information criteria such as the extended Bayesian information criterion (EBIC) [33], leading to an automatic stopping of the algorithm without the need of additional tuning of the number of boosting iterations. For high-dimensional data with many possible covariates, the computation of the “best” base-learner in each iteration of SubBoost is too costly since base-learners can consist of multiple combinations of different variables. Thus, in a second step we extend the method to Random Subspace Boosting (RSubBoost), which incorporates a random preselection of base-learners in each iteration, enabling the computational scalability to high-dimensional settings. Similar randomization ideas have also been recently proposed in the context of component-wise gradient boosting, where significant computational gains with a promising predictive performance have been observed [34]. Finally, we propose a third extension, called Adaptive Subspace Boosting (AdaSubBoost), with an adaptive random preselection of base-learners in each iteration, where the adaptation is motivated by the recently proposed Adaptive Subspace (AdaSub) method [29, 35]. Here, the idea is to focus on those base-learners which—based on the information from the previous iterations—are more likely to be predictive for the response variable.

The performance of the proposed algorithms is investigated in a simulation study and through various biomedical data examples, comparing it with classical $L_2$Boosting as well as with other approaches including twin boosting [31], stability selection [20], the (relaxed) lasso [1–3] and the elastic net [4].

## Methods

### Variable selection in statistical modelling

We consider a linear regression model

1 $$\begin{aligned} \mathbb {E}(Y_i \,|\, \varvec{X}) = \sum _{j=1}^p \beta _j X_{i,j} ,\quad i=1,\ldots , n, \end{aligned}$$

for a continuous response $\varvec{Y}=(Y_1,\ldots ,Y_n)'$ and covariates $X_1,\ldots ,X_p$, whose observed values are summarized in the design matrix $\varvec{X} = (X_{i,j})\in \mathbb {R}^{n \times p}$. For ease of presentation we assume that the covariates and the response have been mean-centered, so that an intercept term can be omitted. Here, $\varvec{\beta }=(\beta _1,\ldots ,\beta _p)'\in \mathbb {R}^p$ denotes the vector of regression coefficients, which one needs to estimate even when the sample size *n* is small in relation to the number of covariates *p*. In practice, one is interested in estimators $\hat{\varvec{\beta }}\in \mathbb {R}^p$ which are sparse in the sense that only a relatively small number of components of $\hat{\varvec{\beta }}$ are nonzero, i.e.

2 $$\begin{aligned} |\hat{S}| = |\{j\in \{1,\ldots ,p\}:~\hat{\beta }_j\ne 0\}| \ll p, \end{aligned}$$

enhancing the interpretability of the resulting model. At the same time, the sparse estimators should minimize the mean squared error of prediction

3 $$\begin{aligned} {\text {MSE}} = \frac{1}{n_{{\text {test}}}}\sum _{i=1}^{n_{{\text {test}}}}(\varvec{x}_{{\text {test}},i}'\hat{\varvec{\beta }} - y_{{\text {test}},i})^2 \,, \end{aligned}$$

where $(\varvec{x}_{{\text {test}},i}, y_{{\text {test}},i})$, for $i=1,\ldots ,n_{{\text {test}}}$, denotes independent test data from the true data-generating distribution.

Table 1 provides a selective overview of different regularization and variable selection methods. In particular, information criteria reflect the inherent trade-off between sparsity and predictive performance. A general family of $\ell _0$-type selection criteria with penalty parameter $\lambda >0$ is given by

4 $$\begin{aligned} \text {GIC}_{\lambda }((\varvec{X},\varvec{y}),S) = n \cdot \log \left( \frac{\Vert \varvec{y} - \varvec{X} \hat{\varvec{\beta }}_S \Vert ^2}{n}\right) + \lambda |S| \,, \end{aligned}$$

for a subset of variables $S\subseteq \{1,\ldots ,p\}$ and observed data $(\varvec{X},\varvec{y})$, where $\hat{\varvec{\beta }}_S\in \mathbb {R}^p$ denotes the least-squares estimator under the linear model (1) with active variables in *S* only, i.e.

5 $$\begin{aligned} \hat{\varvec{\beta }}_S = \mathop {\mathrm{arg min}}\limits _{\varvec{\beta }\in \mathbb {R}^p} \{\left\| \varvec{y} - \varvec{X} \varvec{\beta }\right\| :~ \beta _j = 0 \text { for } j \notin S \}. \end{aligned}$$

The choice of the penalty parameter $\lambda =2$ in $\text {GIC}_{\lambda }$ corresponds to the Akaike information criterion (AIC) [24], while the choice $\lambda = \log (n) + 2\gamma \log (p)$ with constant $\gamma \in [0,1]$ yields the extended Bayesian information criterion (${\text {EBIC}}_\gamma$) [33], with the original BIC [25] as special case for $\lambda =\log (n)$. While minimizing the BIC provides model selection consistency under the classical asymptotic setting (*p* fixed, $n\rightarrow \infty$), minimization of the ${\text {EBIC}}_\gamma$ has been shown to yield model selection consistency under reasonable assumptions for high-dimensional settings ($p,n\rightarrow \infty$) [26, 33]. In general, the identification of the subset *S* which minimizes a particular $\ell _0$-type selection criterion is computationally hard, since the number of possible subsets $S\subseteq \{1,\ldots ,p\}$ grows exponentially with the number of covariates *p*.

Thus, computationally more efficient regularization methods such as the lasso [1] have been developed which make use of the $\ell _1$-norm ($\Vert \varvec{\beta }\Vert _1 = \sum _{j} |\beta _j|$) as a convex relaxation to the “$\ell _0$-norm” ($\Vert \hat{\varvec{\beta }}_S\Vert _0 = |S|$) in (4). On the other hand, several heuristic optimization methods have been proposed to address the combinatorial problem of minimizing $\text {GIC}_{\lambda }$, including different variants of classical stepwise selection [27, 36] as well as stochastic optimization methods such as “Shotgun Stochastic Search” [28] and Adaptive Subspace methods [29, 35].

### Statistical boosting

Statistical boosting is an alternative variable selection approach which is similar to forward stagewise algorithms [5, 37]. In contrast to classical forward selection, boosting leads to a slower overfitting behavior and shrinkage of the estimated coefficients, similarly to regularization methods (such as the lasso).

The classical component-wise $L_2$Boosting algorithm (Algorithm 1) takes the design matrix $\varvec{X}\in \mathbb {R}^{n\times p}$ and the observed continuous response vector $\varvec{y}\in \mathbb {R}^{n}$ as input and, after $m_{{\text {stop}}}$ iterations, yields the estimator $\hat{\varvec{\beta }}^{[m_{{\text {stop}}}]}\in \mathbb {R}^p$ with selected variables in $\hat{S} = \{j:\,\hat{\beta }_j^{[m_{{\text {stop}}}]}\ne 0 \}\subseteq \{1,\ldots ,p\}$ as output. Here, we introduce some additional notation, which will also be convenient in the context of the proposed extensions: in the following let ${{\mathcal {P}}}=\{1,\ldots ,p\}$ denote the index set of covariates $X_1,\ldots ,X_p$. Furthermore, for a subset $S\subseteq {\mathcal {P}}$, let ${\mathcal {P}}{\setminus } S = \{j\in {\mathcal {P}}: j \notin S\}$ denote the difference set and let $\varvec{\beta }_{{\mathcal {P}}{\setminus } S}\in \mathbb {R}^{p-|S|}$ denote the vector $\varvec{\beta }\in \mathbb {R}^p$ restricted to the components in ${\mathcal {P}}{\setminus } S$.
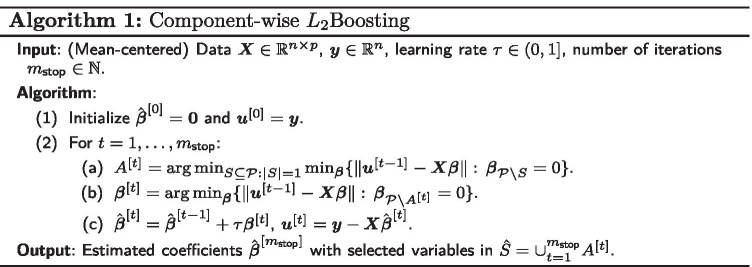


In the first step of $L_2$Boosting, the vector of regression coefficients is initialized as the zero vector, i.e. $\hat{\varvec{\beta }}^{[0]}=\varvec{0}$, and the current vector of residuals is set to the observed response vector, i.e. $\varvec{u}^{[0]}=\varvec{y}$. Then, in each iteration $t=1,\ldots , m_{{\text {stop}}}$ of the algorithm, the “best component” $A^{[t]}$ is selected among all linear component-wise base-learners ($S\subseteq {\mathcal {P}}$ with $|S|=1$), which leads to the best fit of the current residuals $\varvec{u}^{[t-1]}$. Subsequently, the estimated coefficient vector $\hat{\varvec{\beta }}^{[t]} =\hat{\varvec{\beta }}^{[t-1]} + \tau \varvec{\beta }^{[t]}$ is adjusted in the direction $\varvec{\beta }^{[t]}$ of the selected component by the multiplication with a small learning rate $\tau$ (e.g. $\tau =0.1$) and the vector of residuals $\varvec{u}^{[t]} = \varvec{y} - \varvec{X}\hat{\varvec{\beta }}^{[t]}$ is updated. Stopping the algorithm after $m_{{\text {stop}}}$ iterations generally leads to variable selection, since only those variables $X_j$ with $j\in \hat{S} = \cup _{t=1}^{m_{{\text {stop}}}} A^{[t]}$ are included in the final model, which have been selected at least once as the best component.

The stopping iteration $m_{{\text {stop}}}$ is a crucial tuning parameter of $L_2$Boosting, since it controls the induced shrinkage and sparsity. In practice, the choice of $m_{{\text {stop}}}$ is typically guided by optimizing the predictive performance via cross-validation (CV) or bootstrapping techniques. However, in sparse high-dimensional settings, tuning regarding prediction accuracy often yields a final set $\hat{S}$ of selected variables with many false positives (see results below). A simple approach to induce sparser models is the “earlier stopping” of the $L_2$Boosting algorithm, as implemented in the R-package xgboost [38]: the algorithm is stopped as soon as the CV-error does not improve for a particular number of succeeding iterations. This approach can also lead to a reduced computational time, as $L_2$Boosting does not have to be run for a prespecified maximum number of iterations; however, earlier stopping tends to come at the cost of an increase in false negatives and larger shrinkage of effect estimates.

Different extensions of $L_2$Boosting have been proposed to simultaneously reduce the number of selected noise variables and the induced shrinkage. Among them is twin boosting [31], which implements a two-stage approach: the first stage consists of a standard $L_2$Boosting model with tuning of the stopping iteration $m_{1}$, yielding the estimated coefficient vector $\hat{\varvec{\beta }}^{[m_{1}]}$. Then, in the second stage, an additional run of an adjusted $L_2$Boosting algorithm is conducted, where selection step (a) in Algorithm 1 is modified so that components $j\in {\mathcal {P}}$ with large absolute coefficients $|\hat{\beta }_j^{[m_{1}]}|$ from the first stage are updated more frequently in the second stage, reducing the imposed shrinkage for the corresponding variables [31]. After tuning of the stopping iteration $m_{2}$ in the second stage, the final estimated coefficient vector $\hat{\varvec{\beta }}^{[m_{2}]}$ with corresponding set of variables $\hat{S}_{\text {twin}}=\{j\in {\mathcal {P}}:\hat{\beta }_j^{[m_{2}]}\ne 0\}$ is obtained, which is in general a subset of the variables selected by a single run of $L_2$Boosting.

Stability selection is a general ensemble approach to control the number of false positive variables [18]. In the context of boosting [20, 21], stability selection applies a boosting algorithm on several subsamples of size $\left\lfloor n/2\right\rfloor$ from the fully observed data of size *n*. Then, for each variable $X_j$, its relative selection frequency $f_j=\frac{1}{K}\sum _{k=1}^K \mathbbm {1}_{S^{[k]}}(j)$ is computed, where $S^{[k]}$ denotes the variables selected by boosting for the *k*th subsample ($k=1,\ldots ,K$). Finally, for a threshold $\pi _{\text {thr}}\in (0,1)$, the selected set of variables by stability selection is defined by $\hat{S}_{\text {stab}} = \{j\in {\mathcal {P}}: f_j\ge \pi _{\text {thr}}\}$, where the threshold $\pi _{\text {thr}}$ can be chosen in order to control the expected number of false positives (see [18, 19] for details). The idea behind stability selection is to consider only those variables to be “stable” which are selected frequently for different subsamples of the observed data, so that, for a sensible choice of the threshold $\pi _{\text {thr}}$, the model $\hat{S}_{\text {stab}}$ is typically much sparser than the model selected by a single run of boosting for the full dataset.

### Proposed extensions of boosting

We propose three consecutive extensions of $L_2$Boosting with the aim of generating more flexible regularization paths and encouraging sparser solutions. In contrast to twin boosting and stability selection which use multiple runs of the original or slightly adjusted $L_2$Boosting algorithm to yield sparser models, the novel extensions modify the boosting algorithm directly through the choice of base-learners.

#### Subspace Boosting (SubBoost)

We first introduce Subspace Boosting (SubBoost) as a natural extension of $L_2$Boosting (Algorithm 1): additionally to the standard component-wise base-learners, further base-learners can be selected which estimate the effects of multiple variables, implying that coefficients can be updated jointly in a single iteration. However, in order to counterbalance the larger flexibility, the final selection of the components to be updated is based on an additional *double-checking* step via a likelihood-based variable selection procedure. 
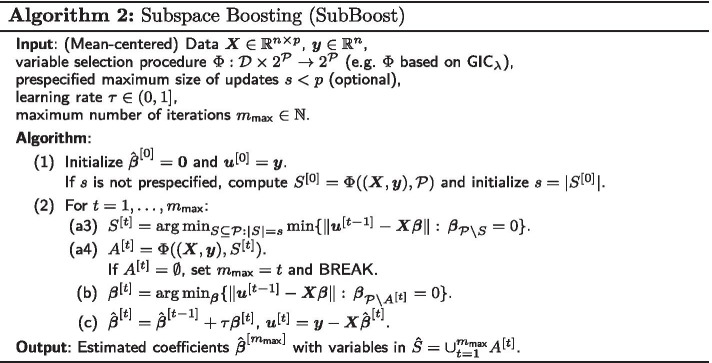


The details of SubBoost are given in Algorithm 2. There are two main differences to classical $L_2$Boosting (Algorithm 1) regarding the selection step (a). First, in step (a3) of SubBoost the “best” subset $S^{[t]}$ of size $|S^{[t]}|=s$ is computed which yields the best fit to the current residuals $\varvec{u}^{[t-1]}$. Here, in contrast to component-wise $L_2$Boosting with $s=1$, the number of components *s* to be updated can be larger than one. Second, in an additional *double-checking* step (a4) we consider a prespecified variable selection procedure $\Phi :{\mathcal {D}} \times 2^{\mathcal {P}} \rightarrow 2^{\mathcal {P}}$, where ${\mathcal {D}}$ denotes the sample space and $2^{\mathcal {P}}=\{S:S\subseteq {\mathcal {P}}\}$ the power set of ${\mathcal {P}}=\{1,\ldots ,p\}$. For a given subset *S* of variables and observed data $(\varvec{X},\varvec{y})\in {\mathcal {D}}$, the selection procedure $\Phi$ yields the model $\Phi ((\varvec{X},\varvec{y}),S)\subseteq S$ with variables selected within *S*. Here, we consider the minimization of likelihood-based $\ell _0$-type generalized information criteria (see Eq. (4)) such as the AIC, the BIC or the EBIC:

6 $$\begin{aligned} \Phi ((\varvec{X},\varvec{y}),S) = \mathop {\mathrm{arg min}}\limits _{A\subseteq S} \text {GIC}_{\lambda }((\varvec{X},\varvec{y}),A) \,. \end{aligned}$$

In step (a4) of SubBoost, $\Phi$ is applied to the “best” set $S^{[t]}$ of *s* variables from step (a3), yielding the final subset $A^{[t]}= \Phi ((\varvec{X},\varvec{y}), S^{[t]}) \subseteq S^{[t]}$ of components to be updated in iteration *t*. Thus, while the maximum size of multivariable updates is given by $|S^{[t]}|=s$, the realized updates $A^{[t]}$ can be of smaller and varying sizes $|A^{[t]}|\le s$ in different iterations *t*. Here, it is important to note that the variable selection procedure $\Phi$ considers the observed data $(\varvec{X},\varvec{y})$ and not the current residuals $(\varvec{X},\varvec{u}^{[t-1]})$ as input data, so that the selection is based on the original likelihood. By this double-checking step it is ensured that variables which would never, for any subset of variables $S\subseteq {\mathcal {P}}$, be selected by the base procedure $\Phi$ on the originally observed data $(\varvec{X},\varvec{y})$, are also not selected in SubBoost even when they may provide the best fit to the current residuals in a particular iteration of the algorithm. Therefore, noise variables are less likely to be selected by SubBoost and the sparsity of the final model is encouraged.

The best model according to $\Phi$ among all considered variables with indices in ${\mathcal {P}}=\{1,\ldots ,p\}$ is given by $A^*=\Phi ((\varvec{X}, \varvec{y}),{\mathcal {P}})$. However, in practice there are often many models $A^{[t]}\subseteq {\mathcal {P}}$ with $A^{[t]}\ne A^*$ of reasonable size which provide a similar fit. Estimating the coefficient vector on the single best model $A^*$ according to $\Phi$ would generally not take into account the model uncertainty (see e.g. [39]). The SubBoost algorithm can be interpreted as a sequential ensemble method, since estimates from multiple “best” models $A^{[t]}=\Phi ((\varvec{X}, \varvec{y}),S^{[t]})$ with $S^{[t]}\subseteq {\mathcal {P}}$ are combined in an adaptive way, where $A^{[t]}$ is the best model according to $\Phi$ when only variables in $S^{[t]}$ are considered. Note that the maximum size of updates $s=|S^{[t]}|$ in SubBoost can be prespecified or, alternatively, be determined by the best model according to $\Phi$, i.e. by computing $S^{[0]}=A^*=\Phi ((\varvec{X}, \varvec{y}),{\mathcal {P}})$ and setting $s=|S^{[0]}|$. The latter option constitutes an effective data-driven way to determine a suitable maximum update size *s* in case of no particular prior information.

A favorable consequence of double-checking with likelihood-based selection criteria is that it can lead to an *automatic stopping* of the SubBoost algorithm: if for some iteration *t* the selected subset $A^{[t]}$ after step (a4) is the empty set, the algorithm is stopped since no components will be updated and the vector of residuals $\varvec{u}^{[t]}=\varvec{u}^{[t-1]}$ will remain the same, leading to the same result also in the following iterations. Note that in data situations where most of the predictor variables are informative, the automatic stopping criterion may not be reached in the sense that $A^{[t]}=\emptyset$ for some iteration *t*; instead the SubBoost algorithm may continue to update the effects of some signal variables with diminishing changes, indicating the convergence of the algorithm. However, this behavior is unlikely in situations with several noise variables, particularly in sparse settings. In all cases, the base variable selection procedure $\Phi$ controls the sparsity of the final model and there is no need for additional tuning of the stopping iteration via resampling methods.

#### Random and Adaptive Subspace Boosting (RSubBoost and AdaSubBoost)

**Fig. 1:**
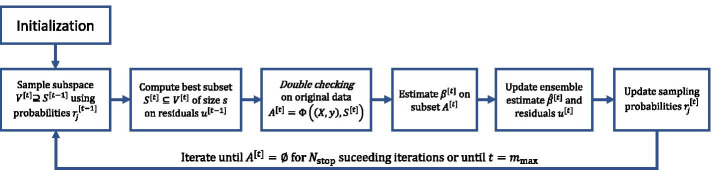
Schematic flowchart of Adaptive Subspace Boosting (AdaSubBoost). For details see Algorithm 3

For high-dimensional data with a large number of variables *p* it can be prohibitive to compute in every iteration the *s* components yielding the best fit to the current residuals in step (a3) of the SubBoost algorithm, since there are $$\left( {\begin{array}{c}p\\ s\end{array}}\right)$$ possible subsets of size *s* which have to be considered. Instead of searching through all possible base-learners of size *s*, it is natural to consider only a random selection of variables for a possible update in each iteration of the algorithm. Thus, we propose two extensions of SubBoost, called Random Subspace Boosting (RSubBoost) and Adaptive Subspace Boosting (AdaSubBoost), which are based on an (adaptive) random preselection of base-learners (see Algorithm 3 and Fig. 1).
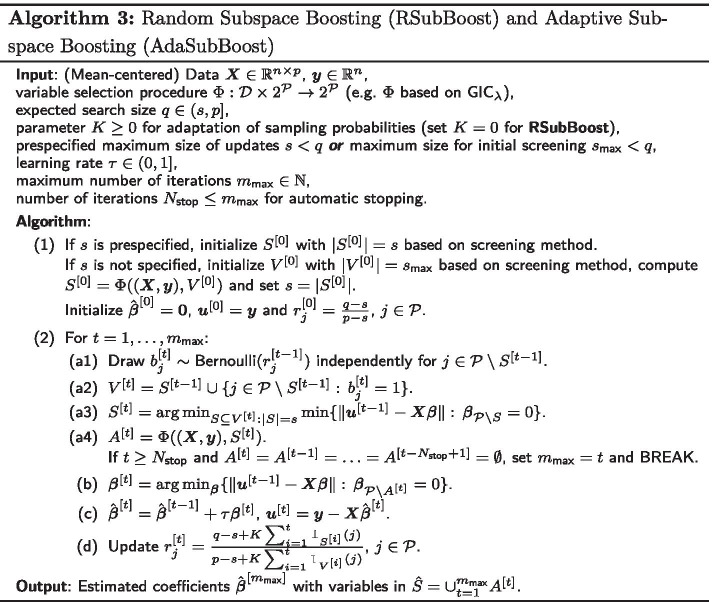


More specifically, the additional steps (a1) and (a2) in Algorithm 3 concern the random preselection of base-learners: in step (a1), independent Bernoulli random variables $b_j^{[t]}\sim \text {Bernoulli}(r_j^{[t-1]})$ with sampling probabilities $r_j^{[t-1]}$ are generated for $j\in {\mathcal {P}}{\setminus } S^{[t-1]}$. Then, in step (a2), the set of variables considered for a possible update in iteration *t* is defined by $V^{[t]}=S^{[t-1]} \cup \{j\in {\mathcal {P}}{\setminus } S^{[t-1]}:\,b_j^{[t]}=1\}$, i.e. $V^{[t]}$ includes all variables in $S^{[t-1]}$ as well as a random set of additional variables (for which $b_j^{[t]}=1$). Here the idea is to reconsider the variables in $S^{[t-1]}$ for a possible update in the next iteration *t*, since they did yield the best fit to the residuals in the previous iteration and are thus likely to be selected again in the next iteration based on the updated residuals. By this, the speed of convergence of the algorithm is increased and the sparsity of the final estimator is encouraged, as variables which have already been updated are more likely to be updated in the succeeding iterations as well. Steps (a3)-(c) in AdaSubBoost are basically the same as for the SubBoost algorithm, while in step (d) the sampling probabilities $r_j^{[t]}$ are adapted based on the currently estimated “importance” of the individual variables $X_j$. Here we employ a similar adaptation rule as in the Adaptive Subspace (AdaSub) method [29]: the sampling probability of variable $X_j$ in iteration $t+1$ is given by

7 $$\begin{aligned} r_j^{[t]}=\frac{q-s+K\sum _{i=1}^t \mathbbm {1}_{S^{[i]}}(j)}{p-s+K\sum _{i=1}^t \mathbbm {1}_{V^{[i]}}(j)} \, , \end{aligned}$$

where $\mathbbm {1}_S$ denotes the indicator function for a set *S*. Thus, $r_j^{[t]}$ can be viewed as a scaled fraction of the number of times variable $X_j$ has been selected in the set $S^{[i]}$ divided by the number of times variable $X_j$ has been considered in the set of possible base-learners $V^{[i]}$, $i\le t$. Therefore, those variables $X_j$, which already yielded a good fit in many previous iterations, are also reconsidered with a larger probability in the set of base-learners for the succeeding iterations of AdaSubBoost.

The Random Subspace Boosting (RSubBoost) algorithm can be regarded as a special case of AdaSubBoost by setting $K=0$, resulting in constant sampling probabilities $r_j^{[t]} = r_j^{[0]}=\frac{q-s}{p-s}$. Thus, in RSubBoost all variables $X_j$ with $j\notin S^{[t-1]}$ have the same probability $P(j\in V^{[t]}) = \frac{q-s}{p-s}$ to be considered in the set of possible base-learners for selection in iteration *t*. In RSubBoost the expectation of the size of $V^{[t]}$ is constant and given by

8 $$\begin{aligned} \mathbb {E}|V^{[t]}|=s + (p-s)\cdot \mathbb {E}\big [b_j^{[t]}\big ] = s + (p-s)\cdot \frac{q-s}{p-s} = q\,, \end{aligned}$$

implying that on average *q* variables are considered for an update in each iteration *t*. The tuning parameter $q\in (s,p]$ controls the expected search size of the algorithm: if *q* is chosen to be small, then only few variables are considered for an update in each iteration; however, if $q=p$ then all variables are always considered, so that the RSubBoost algorithm coincides with the non-randomized SubBoost algorithm. The choice of the expected search size *q* is mainly guided by computational considerations, i.e. *q* should be chosen small enough so that the search step (a3) can be carried out efficiently (e.g. $q\le 25$). On the other hand, *q* should be chosen larger than the maximum update size *s*, so that several “new” variables $X_j$ (with $j\notin S^{[t-1]}$) are considered in the stochastic search. We recommend to use $q=20$ and $s\le 15$, providing computational efficiency and an effective stochastic search (see Additional file 1: Section 2.5.3 for results on the influence of *q*).

The parameter $K\ge 0$ controls the adaptation rate of AdaSubBoost. If *K* is chosen to be large (e.g. $K=10,000$), then the sampling probabilities are adapted quickly; on the other hand, for $K=0$ the RSubBoost algorithm with constant sampling probabilities is retrieved. Regarding the stochastic search for “good” base-learners, *K* controls the trade-off between *exploitation* (corresponding to large *K* with a focus on base-learners which have already proven successful in previous iterations) and *exploration* (corresponding to $K\approx 0$ without a strong focus on particular sets of base-learners). In practice, choosing $K=\frac{p}{q}$ serves as a sensible default in AdaSubBoost (see Additional file 1: Section 2.5.2 for results on the influence of *K*). Note that, regardless of the choice of the sampling probabilities, in each iteration *t* of RSubBoost and AdaSubBoost all variables in $S^{[t-1]}$ (which have provided the best fit to the residuals in the previous iteration) are reconsidered in the subspace $V^{[t]}$ of base-learners. Thus, the adaptive choice of the sampling probabilities only affects the random search in the set of variables ${\mathcal {P}}{\setminus } S^{[t-1]}$ which are additionally considered in the next set of base-learners. In comparison to RSubBoost, the adaptive choice in AdaSubBoost can result in a higher predictive power, as more promising combinations of covariates are considered for potential joint updates. Furthermore, variables $X_j$, which have already been selected, are generally more likely to be updated in the following iterations as well, which further encourages sparsity.

**Fig. 2:**
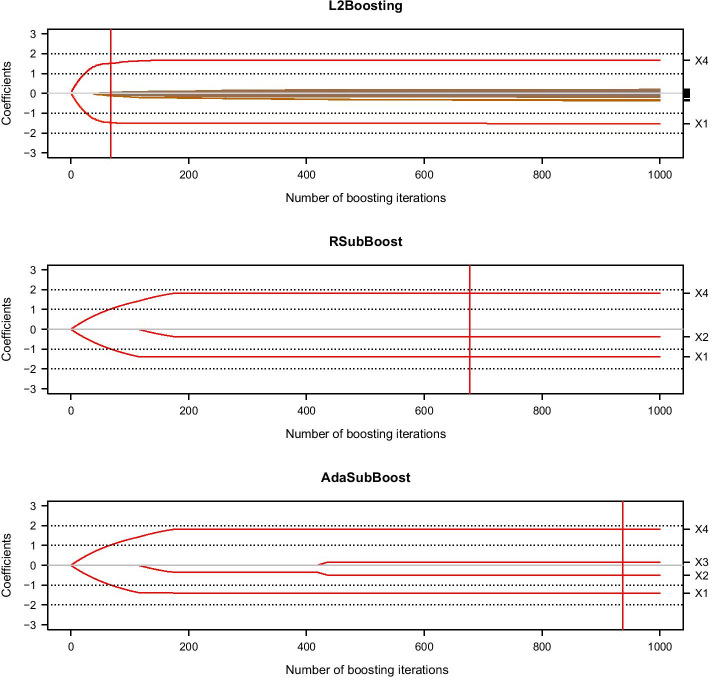
High-dimensional illustrative data example. Coefficient paths $\beta _j^{[t]}$ for $j\in {{\mathcal {P}}}$ along the number of iterations *t* of $L_2$Boosting, RSubBoost and AdaSubBoost. Horizontal black dotted lines indicate the component values of the true $\varvec{\beta }$. For $L_2$Boosting, the vertical red line indicates the CV-optimal stopping iteration $m_{{\text {CV}}}$, while for RSubBoost and AdaSubBoost the automatic stopping after the first $N_{{{\text {stop}}}}=p/2=500$ succeeding iterations without any updates is indicated

Due to the adaptive model building nature of boosting it is crucial that the first iteration of AdaSubBoost (and RSubBoost) starts with a reasonable set of candidate variables $V^{[1]}$, since otherwise uninformative variables may be selected, which would not have been selected if other informative variables had already been considered in $V^{[1]}$. Thus, a screening method such as component-wise $L_2$Boosting (Algorithm 1), forward regression [36] or sure independence screening based on marginal associations [40] should be applied to select an initial set $S^{[0]}$ of $|S^{[0]}|=s$ variables (in case the maximum update size *s* is prespecified). Alternatively and similarly as in the SubBoost algorithm, the maximum update size *s* can be selected in a data-driven way, by first screening a subset $V^{[0]}$ of size $|V^{[0]}|=s_{{\text {max}}}$ (e.g. $s_{{\text {max}}}=15$), computing the best model $S^{[0]}=\Phi ( (\varvec{X},\varvec{y}), V^{[0]})$ according to $\Phi$ restricted to variables in $V^{[0]}$ and setting $s=|S^{[0]}|$. Since $S^{[0]}\subseteq V^{[1]}$ by the construction of the algorithm, all screened variables in $S^{[0]}$ will be considered for an update in the first iteration of AdaSubBoost. If not indicated otherwise, in this work we will use forward regression in the initial screening step and apply the data-driven approach for selecting the maximum update size *s* (except for the two simulation examples in Figure 2 and Additional file 1: Fig. S1, where we prespecify $s=2$ for illustration purposes). Note that AdaSubBoost and RSubBoost also provide automatic stopping similarly to SubBoost. However, the algorithms should not be stopped immediately when $A^{[t]}=\emptyset$, since in the following iterations $t'>t$ with different random sets $V^{[t']}$ the selected sets $S^{[t']}$ and $A^{[t']}$ may change again. In practice, the algorithms may be stopped before the maximum number of iterations $m_{{\text {max}}}$ is reached, if no variables are updated for a prespecified number of iterations $N_{{\text {stop}}}$ (e.g. $N_{{\text {stop}}}=\frac{p}{s}$), i.e. the algorithms are stopped at iteration $t\ge N_{{\text {stop}}}$ if $A^{[t]}=A^{[t-1]}=\cdots =A^{[t-N_{{\text {stop}}}+1]}=\emptyset$.

**Table 2:**
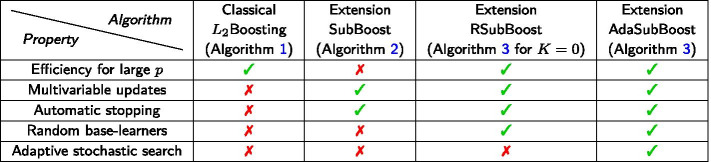
Comparison of classical component-wise $L_2$Boosting with the three proposed extensions: Subspace Boosting (SubBoost), Random Subspace Boosting (RSubBoost) and Adaptive Subspace Boosting (AdaSubBoost)

Table 2 provides a compact overview regarding the properties of component-wise $L_2$Boosting and the novel extensions SubBoost, RSubBoost and AdaSubBoost. In contrast to component-wise $L_2$Boosting, all three extensions allow multivariable updates of effects in a single iteration, as well as double-checking steps with a likelihood-based variable selection procedure $\Phi$, providing automatic stopping of the algorithms and enhanced sparsity. The randomized preselection of base-learners in RSubBoost and AdaSubBoost leads to efficient algorithms even in high-dimensional settings with a large number of covariates *p*, with AdaSubBoost additionally providing an adaptive stochastic search in the space of base-learners based on the information from all previous iterations. An R package implementing the three proposed subspace boosting algorithms is available at GitHub (https://github.com/chstaerk/SubBoost).

## Results

The particular differences between classical component-wise $L_2$Boosting and the proposed randomized extensions RSubBoost and AdaSubBoost are first investigated based on an illustrative high-dimensional simulated data example. Then, a systematic simulation study is conducted in which the predictive performance and variable selection properties of the new algorithms are analyzed in comparison to competing boosting and regularization methods. Finally, the performance of the different methods is compared for various biomedical data applications.

### Illustrative high-dimensional example

An illustrative high-dimensional dataset is simulated according to the linear regression model (1) with $p=1000$ covariates, $n=100$ samples, standard normally distributed errors and sparse coefficient vector $\varvec{\beta }=(-2,-1,1,2,0,\ldots ,0)'\in \mathbb {R}^p$, i.e. only variables $X_1,X_2,X_3$ and $X_4$ are informative for the response *Y*. Furthermore, samples of continuous covariates are independently generated from a multivariate normal distribution with a Toeplitz correlation structure, i.e. $\varvec{x}_i\sim \mathcal {N}_p(\varvec{0}, \varvec{\Sigma })$ for $i=1,\ldots ,n$ with covariance matrix entries $\Sigma _{j,k}=\rho ^{|j-k|}$. The correlation between adjacent covariates is set to $\rho =0.8$, representing a challenging but realistic scenario.

The performance of $L_2$Boosting is illustrated in Figure 2, where the coefficient paths along the number of iterations are shown for the high-dimensional data example (using the R-package mboost [7]). The “optimal” stopping iteration $m_{\text {CV}}$ selected by 10-fold cross-validation (CV) implies that several components corresponding to noise variables are included in the $L_2$Boosting model after $m_{\text {CV}}$ iterations. In particular, the CV-optimal stopping iteration results in an estimate $\hat{\varvec{\beta }}^{[m_{\text {CV}}]}$ with $|\{j\in {\mathcal {P}}:\,\hat{\beta }_j^{[m_{\text {CV}}]}\ne 0 \}| = 14$ non-zero components (selected variables), among which 12 are false positives (i.e. $j\in \{5,\ldots ,p\}$) while only two are true positives (i.e. $j\in \{1,\ldots ,4\}$). Thus, the CV-optimal $L_2$Boosting model yields an unnecessarily large number of selected variables and also misses the two correlated signal variables $X_2$ and $X_3$ with opposite effects on the response.

To illustrate the performance of the subspace boosting algorithms, we apply RSubBoost and AdaSubBoost on the simulated dataset using the ${\text {EBIC}}_\gamma$ with $\gamma =1$ in the selection procedure $\Phi$, which is particularly suitable for high-dimensional data (cf. [33]). In contrast to component-wise $L_2$Boosting (which implicitly is restricted to $s=1$), the number of components to be updated in the subspace algorithms is set to $s=2$. In all subspace algorithms we use the “leaps-and-bounds” algorithm implemented in the R-package leaps [41] for computing the best subsets in steps (a3) and (a4) of the algorithms. While in $L_2$Boosting the default learning rate $\tau =0.1$ is used, in the subspace algorithms the learning rate is set to $\tau =0.01$; note that, due to the stochastic nature of RSubBoost and AdaSubBoost considering only a random subspace of all base-learners in each iteration, it is generally recommended to choose a relatively small learning rate, so that the estimated effects of important covariates are more likely to be updated multiple times in combination with various other important covariates. The mean number of covariates in RSubBoost and AdaSubBoost considered for a possible update in each iteration is initialized as $q=10$, while $K=\frac{p}{q}$ is used as the adaptation parameter in AdaSubBoost. Since the application of SubBoost is computationally intractable for high-dimensional search spaces, we only compare the performance of its randomized extensions with classical $L_2$Boosting (see Additional file 1: Section 1 for an illustrative low-dimensional example including SubBoost).

Figure 2 illustrates that no false positives are included in the RSubBoost and AdaSubBoost models, as the double-checking with ${\text {EBIC}}_1$ prevents the selection of such variables in this case. In contrast to $L_2$Boosting, the signal variable $X_2$ is selected by RSubBoost as it is jointly updated with the correlated variable $X_4$ (having an opposite effect on the response); this illustrates the potential benefits of considering multivariable base-learners. Note that RSubBoost induces somewhat less shrinkage on the effect estimate for $X_4$ in comparison to $L_2$Boosting. While RSubBoost does not select variable $X_3$, the adaptive choice of the sampling probabilities in AdaSubBoost leads to the detection of the signal variable $X_3$. In order to analyze this favorable behavior, it is instructive to investigate the realized joint updates $A^{[t]}$ along the iterations of RSubBoost and AdaSubBoost: during the first iterations of both algorithms (using the same random seed), variables $X_1$ and $X_4$, having the largest effects on the response, are updated jointly ($A^{[t]}=\{1,4\}$ for $t=1,\ldots ,115$). Subsequently, variables $X_2$ and $X_4$ are also updated together ($A^{[t]}=\{2,4\}$ for $t=116,\ldots ,166$). The RSubBoost algorithm does not select any further variables and the stopping criterion is reached after 677 iterations. However, since variables $X_1$ and $X_2$ have already been updated several times, their sampling probabilities $r_1^{[t]}$ and $r_2^{[t]}$ have been increased in AdaSubBoost, so that they are more likely to be reconsidered in the following iterations. This adaptation finally enables AdaSubBoost to identify the beneficial joint updates of variables $X_1$ and $X_3$ ($A^{[419]}=\{1,3\}$) as well as of variables $X_2$ and $X_3$ ($A^{[t]}=\{2,3\}$ for $t=420,\ldots ,437$). Subsequently, no further updates occur ($A^{[t]}=\emptyset$ for $t\ge 438$), so that AdaSubBoost reaches the stopping criterion after 937 iterations. Thus, AdaSubBoost is the only algorithm which identifies the true underlying model $S_{{\text {true}}}=\{1,2,3,4\}$ for this setting.

**Fig. 3:**
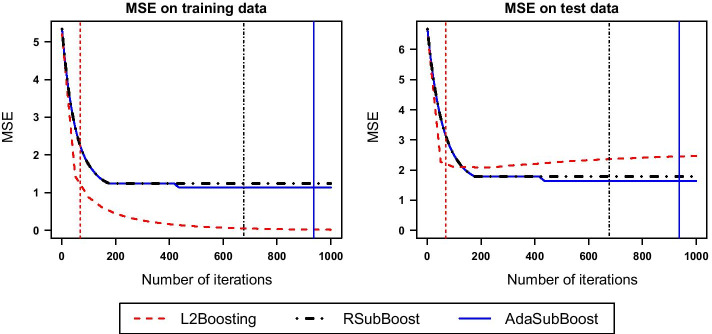
Prediction error for high-dimensional illustrative data example. Mean squared error (MSE) of prediction on training data and independent test set (of size 1000), along the number of iterations of $L_2$Boosting, RSubBoost and AdaSubBoost (cf. Fig. 2). The vertical lines indicate the stopping iterations of the algorithms

The favorable estimation and variable selection properties of RSubBoost and AdaSubBoost also imply an improvement in predictive performance (see Figure 3). In contrast to $L_2$Boosting, the MSE on the training data for the subspace algorithms does not decline towards zero as the number of iterations increases; instead, RSubBoost and AdaSubBoost induce an automatic stopping of learning. While classical $L_2$Boosting continues to improve the fit to the training data, leading to a worsening performance on test data, the new extensions do not suffer from overfitting. In this example, AdaSubBoost yields the smallest prediction error on test data, as it is the only method which exactly identifies the true model.

### Simulation study

#### Low-dimensional setting

In this simulation study we first examine a low-dimensional setting with $p=20$ candidate variables (cf. Additional file 1: Section 1 for an illustrative low-dimensional example). As in the illustrative high-dimensional example, we consider $n=100$ samples, multivariate normally distributed covariates using a Toeplitz correlation structure with $\rho =0.8$ and the true model $S_{{\text {true}}}=\{1,2,3,4\}$; however, to examine a variety of settings, for each of 500 different simulated datasets (simulation replicates), the true coefficients $\beta _j$ for $j\in S_{{\text {true}}}$ are not the same but independently simulated from the uniform distribution $U(-2,2)$. Since we are facing a low-dimensional setting, the standard BIC is used in the selection procedure $\Phi$ for the subspace algorithms. Further parameters in the boosting algorithms are specified as before, except that we do not use a prespecified maximum update size ($s=2$); instead, for each dataset the employed model selection procedure based on the BIC yields the initial selected set $S^{[0]}$ and automatically determines the maximum size $s=|S^{[0]}|\le s_{{\text {max}}}=7$ of the following updates in the subspace boosting algorithms.

To put the results into perspective, we consider $L_2$Boosting [5], twin boosting [31], stability selection [20], the lasso [1], the elastic net [4] and the relaxed lasso [2, 3] as benchmark competitors (see Table 1). For $L_2$Boosting (Algorithm 1) we consider two implementations of the algorithm differing in the choice of the stopping iteration: in the first implementation based on the R-package mboost [7], the stopping iteration is chosen by minimizing the 10-fold CV-error within a prespecified maximum number of iterations (here $m_{{\text {max}}}=1000$); in the second implementation based on the R-package xgboost [38], the algorithm is stopped before $m_{{\text {max}}}=1000$ iterations in case the 10-fold CV-error does not improve for a certain number of succeeding iterations (here earlier stopping after 10 iterations without improvements). In both implementations of $L_2$Boosting we set the learning rate to $\tau =0.1$ and consider component-wise linear base-learners (corresponding to a coordinate descent algorithm, by using the options booster="gblinear", updater="coord_descent" and top_k=1 in xgboost [38]). The R-package bst [42] is used for twin boosting, where the optimal stopping iteration is determined via 10-fold CV, the learning rate is set to $\tau =0.1$ and the option twintype=1 is specified (i.e. weights in the second round of boosting are based on the magnitude of estimated coefficients from the first round). The R-package stabs [43] is used for stability selection in combination with classical $L_2$Boosting, where $q_{\text {stab}} = 10$ variables are selected for each subsample and the expected number of selected false positives (i.e. the per-family error rate) is bounded by $\text {PFER}=2$. Classical least squares estimation is used for the final model from stability selection. For all boosting algorithms, the maximum number of iterations is $m_{{\text {max}}}=1000$ in the low-dimensional setting, while RSubBoost and AdaSubBoost incorporate automated stopping after $\frac{p}{2}=10$ succeeding iterations without any updates. The R-package glmnet [44] is used for the lasso and the relaxed lasso, while the additional R-package glmnetUtils [45] is used for tuning the additional parameter $\alpha$ in the elastic net. Final lasso, relaxed lasso and elastic net estimates are based on minimizing the 10-fold CV-error. For comparability reasons, we use serial implementations of all algorithms, without potential parallelization of resampling methods (reported computation times are based on a 2.7GHz processor).

**Fig. 4:**
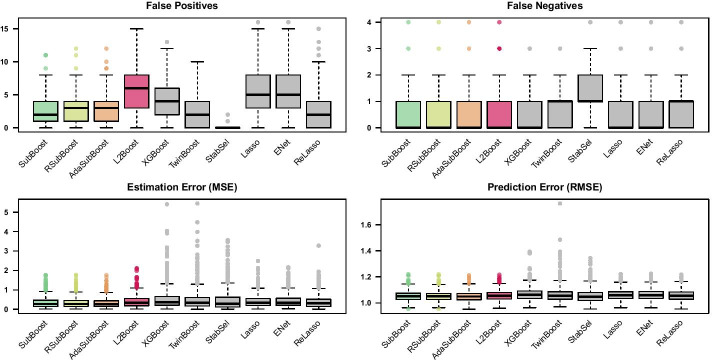
Results for low-dimensional simulation setting. Boxplots of false positives, false negatives, estimation error and prediction error on test set (of size 1000), for 500 simulation replicates with $n=100$, $p=20$, $S_{{{\text {true}}}}=\{1,2,3,4\}$ and Toeplitz correlation with $\rho =0.8$

Figure 4 shows that the three subspace methods SubBoost, RSubBoost and AdaSubBoost systematically reduce the number of false positives in comparison to classical $L_2$Boosting, while the number of false negatives is unaffected (see Additional file 1: Section 2.2 for detailed numerical results). The beneficial variable selection properties lead to small reductions in mean squared errors (MSEs) for estimating the coefficient vectors $\varvec{\beta }\in \mathbb {R}^p$ and in root mean squared errors (RMSEs) of prediction on independent test data. The three subspace boosting algorithms perform very similar in this low-dimensional setting, with AdaSubBoost showing a slightly improved estimation and prediction performance. Earlier stopping of $L_2$Boosting via XGBoost leads to a reduction of false positives, yielding a worse predictive performance in this setting. The competing two-stage twin boosting algorithm also reduces the number of false positives in comparison to the single-stage $L_2$Boosting algorithm; however, the number of false negatives tends to be slightly larger compared to $L_2$Boosting and the subspace boosting algorithms. Stability selection yields very small numbers of false positives, while paying a price in terms of increased numbers of false negatives. Although the average estimation and prediction performance of the sparse models selected by twin boosting and stability selection seem not to be largely affected in this low-dimensional setting with only four informative variables, an increased variability over the different simulation replicates is apparent in comparison to the other boosting methods. The lasso and the elastic net perform similar to $L_2$Boosting (cf. [15]), including larger numbers of noise variables compared to the subspace boosting algorithms. The relaxed lasso tends to yield smaller numbers of false positives than the lasso, but at the cost of increased numbers of false negatives.

#### Sparse high-dimensional settings

Next, we extend the high-dimensional illustrative example from above (see Figures 2 and 3): for 500 simulation replicates, we consider $n=100$ samples, $p=1000$ multivariate normally distributed covariates using a Toeplitz correlation structure with $\rho =0.8$ and true coefficients $\beta _j\sim U(-2,2)$ for $j\in S_{{\text {true}}}$. Here, we examine two sparse high-dimensional settings which differ only in the true underlying models $S_{{\text {true}}}$: in setting (a), the true model $S_{{\text {true}}}=\{1,\ldots ,10\}$ is fixed, while in setting (b) the true model $S_{{\text {true}}}\subset \{1,\ldots ,p\}$ is randomly chosen with $|S_{{\text {true}}}|=10$ for each simulation replicate. While setting (a) in conjunction with the Toeplitz correlation structure implies that high correlations predominantly occur among signal variables ($X_1,\ldots ,X_{10}$), setting (b) induces high correlations mostly between signal and noise variables, as the 10 signal variables are randomly distributed among the $p=1000$ covariates.

In the sparse high-dimensional settings, the ${\text {EBIC}}_1$ is considered in the model selection procedure $\Phi$ and is also used for the initialization of the maximum update sizes $s\le s_{{\text {max}}}=15$ in RSubBoost and AdaSubBoost (see Additional file 1: Figure S4 for additional information on the selected “baseline” models $S^{[0]}$), considering the expected search size $q=20$ and the adaptation parameter $K=\frac{p}{q}$. We refer to Additional file 1: Section 2.5 for sensitivity analyses regarding the choice of the selection procedure $\Phi$ and further tuning parameters $s_{{\text {max}}}$, *q* and *K*. The maximum number of iterations is set to $m_{{\text {max}}}=5000$, while RSubBoost and AdaSubBoost are automatically stopped after $\frac{p}{2}=500$ succeeding iterations without any updates. The remaining parameters for the algorithms are specified as in the low-dimensional setting, except for stability selection where $q_{\text {stab}} = 15$ variables (instead of $q_{\text {stab}} = 10$) are selected for each subsample.

**Fig. 5:**
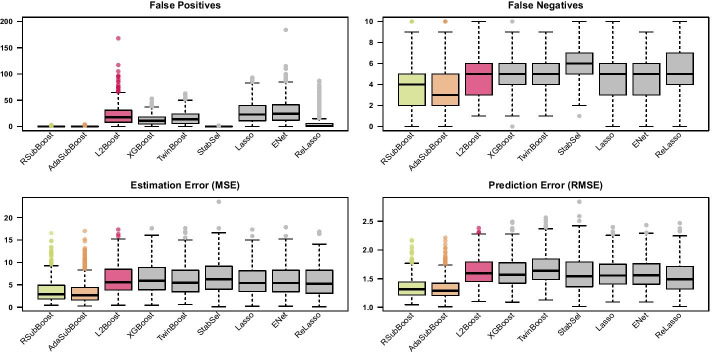
Results for sparse high-dimensional simulation setting (a). Boxplots of false positives, false negatives, estimation error and prediction error on independent test set (of size 1000), for 500 simulation replicates with $n=100$, $p=1000$, $S_{{\text {true}}}=\{1,\ldots ,10\}$ and Toeplitz correlation with $\rho =0.8$

Figure 5 shows that RSubBoost and AdaSubBoost largely reduce the number of false positives in comparison to classical $L_2$Boosting in high-dimensional setting (a). Remarkably, at the same time, the subspace algorithms also tend to yield smaller numbers of false negatives. Figure 5 further indicates an excellent estimation and prediction performance of the subspace boosting algorithms, with slight advantages for AdaSubBoost. These results confirm the observations in the high-dimensional illustrative example discussed above (see Figures 2 and 3): the joint updates of effect estimates in the subspace algorithms are particularly beneficial in cases of high correlations among signal variables; furthermore, in such cases the adaptive selection of base-learners in AdaSubBoost can lead to a higher predictive power. Due to the earlier stopping, XGBoost yields less false positives and more shrinkage of effect estimates than classical $L_2$Boosting, resulting in slightly favorable predictions but a worse estimation performance. Earlier stopping via XGBoost also leads to a considerable reduction of computation times in this sparse setting (see Additional file 1: Table S2 and Figure S3). For twin boosting and even more for stability selection, the reduction in the number of false positives leads to a loss of statistical power for detecting signal variables, so that no systematic improvements in predictive performance over classical $L_2$Boosting are observed. The lasso and the elastic net perform again similar to $L_2$Boosting, yielding relatively large numbers of false positives. The relaxed lasso shows an improved variable selection and prediction performance compared to the classical lasso, but is outperformed by the subspace boosting algorithms in this sparse and highly-correlated setting.

Results for the additional sparse high-dimensional setting (b) with high correlations predominantly between signal and noise variables show that the subspace boosting algorithms again substantially reduce the number of false positives compared to $L_2$Boosting, while providing a competitive predictive performance; however, in contrast to setting (a) with high correlations among signal variables, this comes at the cost of an increase in false negatives. Detailed results for simulation setting (b) can be found in Additional file 1: Section 2.1, while details on computation times for the different simulation settings are provided in Additional file 1: Sections 2.2 and 2.3.

#### Non-sparse high-dimensional setting

Finally, we consider a non-sparse setting, where the true model $S_{{\text {true}}}=\{1,\ldots ,100\}$ is fixed and consists of 100 signal variables (out of $p=1000$ candidate variables), while the sample size is $n=1000$. In the non-sparse setting we additionally consider the AIC as an alternative selection procedure $\Phi$, inducing less sparsity than the ${\text {EBIC}}_1$. The maximum number of iterations is set to $m_{{\text {max}}}=10,000$ in the different boosting algorithms, while we set $q_{\text {stab}} = 150$ in stability selection. The remaining parameters for the algorithms and further simulation specifications are the same as in the sparse high-dimensional settings.

**Fig. 6:**
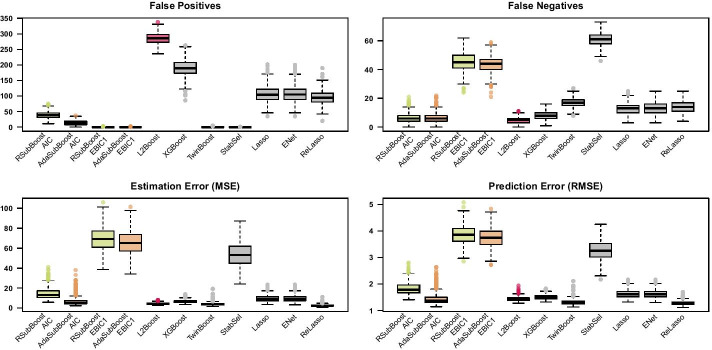
Results for the non-sparse high-dimensional simulation setting. Boxplots of false positives, false negatives, estimation error and prediction error on independent test set (of size 1000), for 500 simulation replicates with $n=100$, $p=1000$, $S_{{\text {true}}}=\{1,\ldots ,100\}$ with $|S_{{\text {true}}}|=100$ and Toeplitz correlation with $\rho =0.8$

Figure 6 shows that AdaSubBoost in combination with the ${\text {EBIC}}_1$ yields very small numbers of false positives but large numbers of false negatives, leading to a poor predictive performance in this non-sparse setting. When the AIC is used instead of the ${\text {EBIC}}_1$ for the double-checking in AdaSubBoost, the number of false negatives is reduced, leading to a reasonable predictive performance; however, this comes at the cost of an increase in the number of false positives. Particularly in this non-sparse setting with many informative variables, the adaptive stochastic search in AdaSubBoost is beneficial compared to RSubBoost, yielding less false positives and improved predictions. $L_2$Boosting yields very large models with many false positives, but a competitive predictive performance. Earlier stopping via XGBoost results in a reduction of false positives, but larger numbers of false negatives and a worse prediction performance. In such non-sparse settings, the earlier stopping approach is also not beneficial in terms of computation times (see Additional file 1: Table S4 and Fig. S3). Stability selection yields sparse models with almost no false positives but many false negatives, resulting in a low prediction accuracy. Twin boosting also selects small numbers of false positives, but shows a very good predictive performance in this non-sparse setting, even though several signal variables are not selected. The regularization methods lasso, elastic net and relaxed lasso show a similar variable selection performance with many false positives, while the relaxed lasso yields the best predictive performance in this situation, which is in line with a recent comparative simulation study of Hastie et al. [3]. In summary, this non-sparse setting further illustrates the inherent trade-off between variable selection and predictive performance.

### Applications on biomedical data

**Fig. 7:**
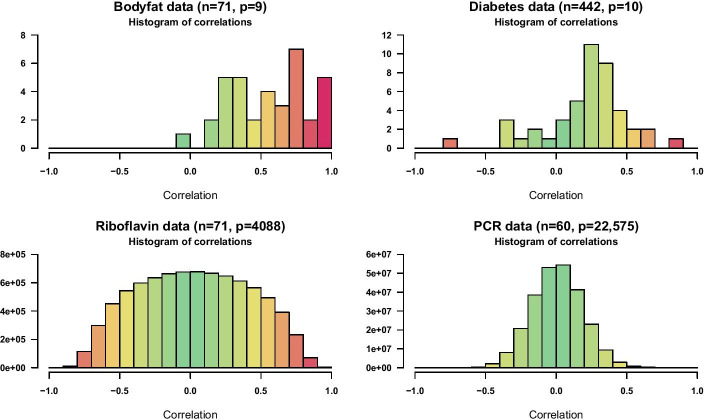
Correlation structure of biomedical datasets. Histograms of pairwise Pearson correlations between the covariates for the two low-dimensional (upper row) and the two high-dimensional datasets (lower row)

In order to evaluate the performance of the proposed subspace boosting algorithms in non-artificial data situations, we examine two low-dimensional and two high-dimensional biomedical datasets, which are publicly available and have previously been investigated using different variable selection methods. In particular, as the first low-dimensional dataset, we consider bodyfat data [46], consisting of body fat measurements for $n=71$ healthy females as the response variable of interest and $p=9$ covariates including age and several anthropometric measurements. As the second low-dimensional example, we consider diabetes data [12], where the response is a quantitative measure of disease progression one year after baseline, with $p=10$ baseline covariates measured for $n=442$ diabetes patients. The bodyfat data has already been analyzed using component-wise $L_2$Boosting [5, 7], while the diabetes data has originally been examined using Least Angle Regression (LARS) with discussions also related to boosting and the lasso [12]. As the first high-dimensional dataset, we consider ribovlavin data [47], where the response consists of $n=71$ observations of log-transformed riboflavin production rates and the covariates are given by logarithmic gene expression levels for $p=4088$ genes. As the second high-dimensional example, we consider polymerase chain reaction (PCR) data [48], where the response is given by a particular physiological phenotype for $n=60$ mice and the full set of covariates comprises $p=22{,}575$ gene expression levels. The ribovlavin data has been previously analyzed using stability selection [49], while the PCR data has, among others, been investigated using a Bayesian split-and-merge approach [50] and the Adaptive Subspace (AdaSub) method [29]. Histograms of correlations between the covariates for the four datasets are shown in Fig. 7.

Here, we evaluate the different algorithms based on external leave-one-out cross-validation (LOOCV), i.e. for each $i\in \{1,\ldots ,n\}$ we consider $n-1$ samples as training data $\{1,\ldots ,n\}{\setminus }\{i\}$ and the single sample $\{i\}$ as test data. The variable selection algorithms are applied independently on each of the *n* training subsamples, yielding potentially different models with varying numbers of selected variables. The performance of the algorithms is assessed based on the number of selected variables and the absolute prediction errors on the independent test samples. For the low-dimensional datasets we consider the three subspace boosting algorithms SubBoost, RSubBoost and AdaSubBoost in combination with the classical BIC, while for the high-dimensional datasets we consider the two randomized algorithms RSubBoost and AdaSubBoost in combination with the ${\text {EBIC}}_1$. The maximum number of iterations in the subspace algorithms is set to $m_{{\text {max}}}=1000$ for the two low-dimensional datasets, while we use $m_{{\text {max}}}=10{,}000$ for the two high-dimensional datasets. Similarly to the simulation study, the parameters in the subspace boosting algorithms are set to $q=\min \{20,p/2\}$ and $K=\frac{p}{q}$ for all four datasets, while we specify $s_{{\text {max}}}=4$ for the low-dimensional and $s_{{\text {max}}}=15$ for the high-dimensional datasets. For the PCR data, instead of forward regression, we apply sure independence screening [40] as a computationally more efficient initial screening step in RSubBoost and AdaSubBoost, which is based on ranking the marginal correlations between the individual covariates and the response. For stability selection, the number of variables selected for the subsamples is set to $q_{\text {stab}}=\min \{15,\lfloor p/2 \rfloor \}$, with $\text {PFER}=2$ as the bound on the expected false positives. All remaining parameters of the competing algorithms are specified as in the simulation study.

For all considered datasets, the computational costs for the proposed subspace algorithms are comparable to classical $L_2$Boosting using the R-package mboost [7] (mean computation times for AdaSubBoost between 1.5 s for bodyfat data and 190 s for PCR data; for $L_2$Boosting between 0.6 s and 114 s). The earlier stopping approach via XGBoost yields reduced computation times particularly in sparse high-dimensional settings (mean of 3 s for PCR data). On the other hand, twin boosting and stability selection tend to be more costly than RSubBoost and AdaSubBoost (means for twin boosting between 11 s and 405 s; for stability selection between 10 s and 312 s). Regularization methods including the (relaxed) lasso an d the elastic net are very efficient using the R-package glmnet [44] (means for lasso between 0.1 s and 1.9 s; for elastic net between 0.7 s and 24 s). We refer to Additional file 1: Section 3 for detailed results on computation times.

**Fig. 8:**
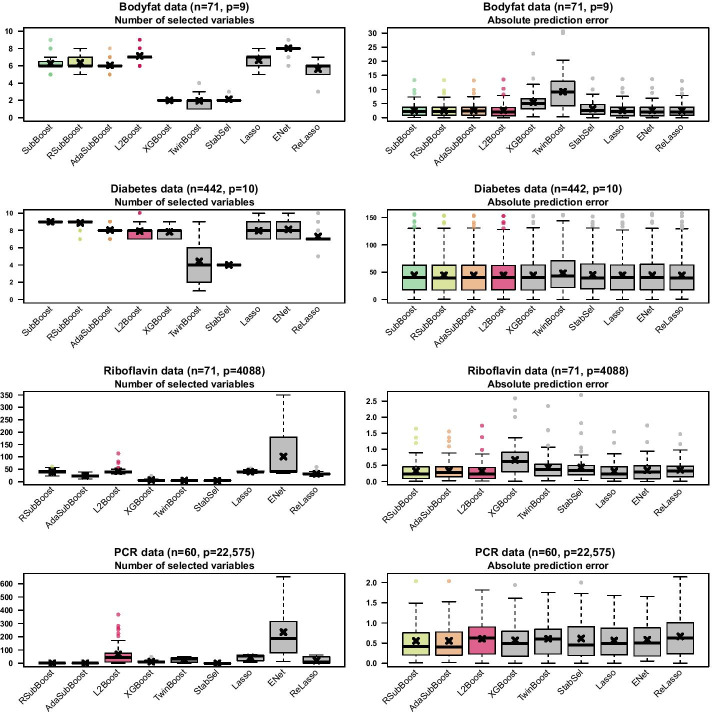
Results for different biomedical applications. Boxplots of numbers of selected variables and absolute prediction errors on out-of-sample data using external leave-one-out cross-validation (LOOCV). Empirical means are depicted by black crosses

Figure 8 shows the results of the different algorithms for external LOOCV applied to the four biomedical datasets (see Additional file 1: Table S5 for detailed numerical results). For the low-dimensional bodyfat data, the three subspace boosting algorithms and classical $L_2$Boosting perform similar, with the subspace algorithms yielding slightly sparser models (all with a median of six selected variables) in comparison to $L_2$Boosting (median of seven variables). SubBoost and RSubBoost perform almost identically for the bodyfat data, while AdaSubBoost tends to select slightly less variables with a competitive predictive performance. The earlier stopping approach via XGBoost, twin boosting and stability selection produce very sparse models in this application with median model sizes of two variables, but lead to a lower prediction accuracy, particularly for twin boosting. The regularization methods lasso, elastic net and relaxed lasso perform quite similar for this dataset, with the elastic net yielding slightly larger models and the relaxed lasso slightly sparser models. For the low-dimensional diabetes data with a larger sample size ($n=442$), the results of SubBoost and RSubBoost are almost equivalent (both with a median of nine selected variables), while AdaSubBoost yields again slightly sparser models (median of eight variables). The predictive performance of the three subspace boosting algorithms is comparable to $L_2$Boosting and to the earlier stopping approach via XGBoost with median model sizes of eight variables. Twin boosting and stability selection reduce the number of selected variables but lead to lower prediction accuracy. It is notable that, in contrast to stability selection and the subspace algorithms, twin boosting yields a larger variability regarding the number of selected variables as well as the lowest prediction accuracy for the two low-dimensional datasets. For the diabetes data, the lasso and the elastic net perform again similar to $L_2$Boosting. In this case, the relaxed lasso yields slightly sparser models than AdaSubBoost with a competitive predictive performance.

Regarding the two high-dimensional riboflavin and PCR datasets, Figure 8 shows that $L_2$Boosting results in relatively large models, with median model sizes of 39 variables for the riboflavin data and 44 variables for the PCR data. For the riboflavin data, RSubBoost yields quite similar model sizes to $L_2$Boosting (median 40 selected variables) with a comparable predictive performance, while AdaSubBoost results in considerably sparser models (median 23 variables). Earlier stopping via XGBoost yields sparser models (median five variables) with a poor predictive performance. Similarly, twin boosting and stability selection yield median model sizes of only four variables, but at the cost of a significant increase in prediction errors. On the other hand, the prediction performance of the relatively sparse AdaSubBoost models is only slightly worse in comparison to $L_2$Boosting. For the riboflavin data, the lasso performs again similar to $L_2$Boosting, while the elastic net results in very unstable variable selection with large numbers of selected variables; the relaxed lasso tends to select more variables (median 31 variables) than AdaSubBoost without beneficial effects on the predictive performance. For the PCR data, $L_2$Boosting, XGBoost, twin boosting, the lasso, the elastic net and the relaxed lasso tend to yield larger models (median model sizes ranging from 8 variables for the relaxed lasso to 186 variables for the elastic net), resulting in a poor predictive performance due to overfitting for this high-dimensional dataset with $p=22{,}575$ variables and only $n=60$ samples. In contrast, RSubBoost and AdaSubBoost produce very sparse models for the PCR data with median model sizes of one, while stability selection almost exclusively yields the intercept model. For the PCR data, the subspace boosting algorithms show the best predictive performance.

## Discussion

We have proposed three consecutive extensions of classical statistical boosting [5]. Results from the simulation study and the biomedical applications indicate that the proposed subspace boosting algorithms tend to yield sparser models with a competitive predictive performance compared to classical component-wise $L_2$Boosting. Even though competing approaches like stability selection [20] and twin boosting [31] also produce sparser models, these methods often result in a loss of predictive power, as several signal variables may not be detected. In this context, one should note that the main target of stability selection is the control of the expected number of false positives, while the objective of the subspace boosting algorithms is good predictive performance with final models as sparse as possible. Our results further show that the new algorithms can yield a favorable predictive performance compared to regularization methods like the (relaxed) lasso in sparse high-dimensional situations (e.g. for sparse high-dimensional simulation settings (a) and (b) as well as for the PCR data), while the predictive performance may be affected in less sparse situations (e.g. for the non-sparse simulation setting).

The adaptive stochastic search in AdaSubBoost is particularly beneficial compared to RSubBoost in settings with high correlations among signal variables as well as non-sparse situations. Nevertheless, the performance of RSubBoost and AdaSubBoost is often similar, as the selection of the base-learners in RSubBoost is already “adaptive” in the sense that predictor variables which yielded the best fit to the residuals in a particular iteration are reconsidered in the set of base-learners for the subsequent iteration. While the adaptation scheme in AdaSubBoost (Algorithm 3) is inspired by the AdaSub method [29], there are important differences between these approaches regarding their main objectives. AdaSub aims to identify the *single best model* according to an $\ell _0$-type selection criterion (such as the EBIC) and thus primarily focuses on variable selection in sparse high-dimensional settings. On the other hand, AdaSubBoost aims at achieving a competitive predictive performance by using an *adaptive ensemble of multiple models*, yielding a particular form of model averaging based on $\ell _0$-type criteria. In particular, due to the adaptive model building concept of boosting, the AdaSubBoost algorithm can also be efficiently applied in high-dimensional settings without underlying sparsity (see non-sparse simulation setting), although in such situations the predictive ability of AdaSubBoost may be reduced in comparison to classical $L_2$Boosting.

Our results indicate that the multivariable updates in the subspace boosting algorithms are advantageous in situations with high correlations among predictor variables, which is also in line with previous studies [32]. Indeed, the new subspace boosting algorithms also have parallels to the block-wise boosting (BlockBoost) algorithm proposed by Tutz and Ulbricht (2009, [32]): in each iteration of BlockBoost, multivariable base-learners can be selected by first ordering the covariates according to their current marginal contributions and then conducting a forward search using an adjusted AIC with an additional correlation-based penalty. Although forward regression or sure independence screening can be used in the initialization step of the subspace boosting algorithms, in contrast to BlockBoost our extensions of $L_2$Boosting do not rely on greedy forward searches, but instead yield exact solutions to the problem of computing the best base-learner within the considered subspace in each iteration. Furthermore, while classical $L_2$Boosting, BlockBoost and SubBoost are deterministic algorithms, the randomized extensions RSubBoost and AdaSubBoost rely on stochastic searches in the space of possible base-learners, enabling the efficient application of the algorithms on very high-dimensional data.

Since RSubBoost and AdaSubBoost constitute stochastic algorithms, one may obtain slightly different results when they are run multiple times on the same dataset. Nevertheless, our results for external leave-one-out cross-validation on the four biomedical datasets show that numbers of selected variables remain relatively stable in comparison to $L_2$Boosting and twin boosting. Furthermore, in practice, using cross-validation for tuning the optimal stopping iteration in classical $L_2$Boosting and twin boosting as well as using subsampling for stability selection also lead to a certain stochasticity in the final models. An important benefit of the double-checking steps in the subspace algorithms is that it leads to automatic stopping, so that no additional tuning of the stopping iteration via resampling methods is needed. Instead, the choice of the selection criterion for the double-checking steps controls the sparsity of the final subspace boosting models. Here we have focused on the BIC for low-dimensional cases and the ${\text {EBIC}}_1$ for high-dimensional cases; however, other selection criteria such as the AIC can also be used in the proposed algorithmic framework as illustrated in the non-sparse simulation setting.

The proposed subspace boosting algorithms are also related to the probing approach for boosting [51]. In probing, the originally observed dataset is first augmented with randomly permuted copies of the covariates (so-called “shadow variables”) and then boosting is automatically stopped as soon as the first “shadow variable” is selected. Thus, while classical statistical boosting is tuned to yield the best predictive performance, the tuning of the stopping iteration in probing and the subspace boosting algorithms takes the variable selection into account, without requiring multiple runs of the algorithms. The resulting savings in computational resources are somewhat counterbalanced by the wider augmented data in probing (with twice as many covariates) and by the additional computational time for the double-checking steps in the subspace boosting algorithms. While probing basically alters only the stopping scheme of boosting, important features of the subspace boosting algorithms include the multivariable updates, the randomized selection of base-learners as well as the double-checking steps via likelihood-based information criteria considering only the observed covariates.

Limitations of this work include that we have only considered $L_2$Boosting with linear base-learners. Further research is warranted on extending our subspace boosting algorithms towards generalized linear models (i.e. other loss functions than the $L_2$-loss) as well as non-linear effect estimates (i.e. other types of base-learners such as regression trees, as efficiently implemented in the R-package xgboost [38]). Furthermore, similarly to other data-driven variable selection approaches, the proposed algorithms are primarily designed for relatively sparse settings, where variable selection is beneficial. In case the underlying data generating process is not sparse, the randomized algorithms are still applicable but may result in a reduced predictive performance due to the tendency to favor sparse and interpretable models. While this work focused on high-dimensional settings (i.e. wide data with many variables *p* and small to moderate sample sizes *n*), future work should be targeted at the extension and practical application of the proposed boosting methods to large-scale data (i.e. big data with large *p* *and* large *n*), such as the development of polygenic risk scores based on millions of single nucleotide polymorphisms (SNPs) and hundred thousands of samples [52]. Another general limitation of the statistical boosting framework is that the computation of standard errors and confidence intervals for effect estimates is not straightforward. Future research may investigate the application of permutation tests [53] and other recent advances in post-selection inference [54] for the new extensions of $L_2$Boosting.

## Conclusions

The three proposed subspace boosting algorithms with multivariable base-learners are promising extensions of statistical boosting, particularly suited for data situations with highly-correlated predictor variables. By using (adaptive) stochastic searches in the space of possible base-learners, the randomized versions can be efficiently applied on high-dimensional data. The incorporated double-checking via information criteria induces automatic stopping of the algorithms, promoting sparser and more interpretable prediction models. The proposed algorithms shift the focus from finding the “optimal” ensemble solution regarding prediction accuracy towards finding a competitive prediction model which is as sparse as possible.

## Supplementary Information

**Additional file 1**. The Supplement includes results for an illustrative low-dimensional data example as well as additional results for the simulation study and the biomedical data applications.

## Data Availability

All biomedical datasets are publicly available: the bodyfat data [46] can be loaded via the R-package TH.data, the diabetes data [12] via the R-package lars, the riboflavin data [47] via the R-package hdi and the PCR data [48] can be downloaded from JRSSB Datasets Vol. 77(5), Song and Liang (2015, [50]) at the website https://rss.onlinelibrary.wiley.com/hub/journal/14679868/series-b-datasets/pre_2016a. An R implementation of the proposed algorithms and source code for reproducing all results is available at GitHub (https://github.com/chstaerk/SubBoost).

## Abbreviations

AdaSub: Adaptive subspace method
AdaSubBoost: Adaptive subspace boosting
AIC: Akaike information criterion
BIC: Bayesian information criterion
BlockBoost: Block-wise boosting
CV: Cross-validation
EBIC: Extended Bayesian information criterion
ENet: Elastic net
GAM: Generalized additive model
GAMLSS: Generalized additive models for location, scale, and shape
GIC: Generalized information criterion
LARS: Least angle regression
Lasso: Least absolute shrinkage and selection operator
*L*_2_ Boost: Component-wise *L*_2_ Boosting with mboost
LOOCV: Leave-one-out cross-validation
MSE: Mean squared error
PCR: Polymerase chain reaction
PFER: Per-family error rate
ReLasso: Relaxed lasso
RMSE: Root mean squared error
RSubBoost: Random subspace boosting
SNP: Single nucleotide polymorphism
StabSel: Stability selection
SubBoost: Subspace boosting
TwinBoost: Twin boosting
XGBoost: Extreme gradient boosting (here with component-wise linear base-learners)
