# Supplementary material for: Randomized boosting with multivariable base-learners for high-dimensional variable selection and prediction
Source: BMC Bioinformatics. 2021 Sep 16;22:441. doi: 10.1186/s12859-021-04340-z (PMC8447543; doi:10.1186/s12859-021-04340-z)
Supplement: Supplementary file 1 — Additional file 1. The Supplement includes results for an illustrative low-dimensional data example as well as additional results for the simulation study and the biomedical data applications. [file 12859_2021_4340_MOESM1_ESM.pdf]

Supplement to “Randomized boosting with multivariable base-learners for high-dimensional variable selection and prediction”

Christian Staerk and Andreas Mayr

Department of Medical Biometry, Informatics and Epidemiology, University Hospital Bonn, Germany

1 Illustrative low-dimensional example

Here we investigate a low-dimensional setting, in which SubBoost (Algorithm 2) can be applied efficiently and compared to classical component-wise L_2Boosting (Algorithm 1) as well as to the randomized extensions RSubBoost and AdaSubBoost (Algorithm 3). Similarly as for the illustrative high-dimensional data example in the main document, we consider a simulated dataset from the linear model (1) with $n = 100$ samples, but only $p = 20$ covariates (instead of $p = 1000$), generated from a multivariate normal distribution using a Toeplitz correlation structure with $\rho = 0.8$. As before, we consider standard normally distributed errors and the true coefficient vector $\boldsymbol{\beta} = (-2, -1, 1, 2, 0, \dots, 0)' \in \mathbb{R}^p$, so that the true model $S_{\text{true}} = \{j \in \mathcal{P} : \beta_j \neq 0\} = \{1, 2, 3, 4\}$ again consists of the first four variables only. Since we are facing a low-dimensional setting, the standard BIC is used in the selection procedure Φ for the double-checking steps of SubBoost, RSubBoost and AdaSubBoost. Further hyper-parameters of the algorithms are specified as for the illustrative high-dimensional example in the main document.

Figure S1 depicts the coefficient paths along the number of iterations of L_2Boosting, SubBoost, RSubBoost and AdaSubBoost. The four signal variables $(X_1, \dots, X_4)$ are included in the final models for all of the four algorithms; however, the CV-optimal L_2Boosting model additionally includes nine noise variables. As a consequence of the double-checking steps with the BIC, the new SubBoost, RSubBoost and AdaSubBoost algorithms do not select any noise variables and recover the true underlying model $S_{\text{true}} = \{1, 2, 3, 4\}$. It can be observed that the coefficient paths for the deterministic SubBoost algorithm and the randomized RSubBoost algorithm are very similar, illustrating that the scalable RSubBoost algorithm closely resembles SubBoost in low-dimensional settings. For this example, the coefficient paths of AdaSubBoost are also similar to those of RSubBoost, as the adaptive choice of the sampling probabilities for the base-learners does not have a large effect in this low-dimensional setting with only $p = 20$ covariates.

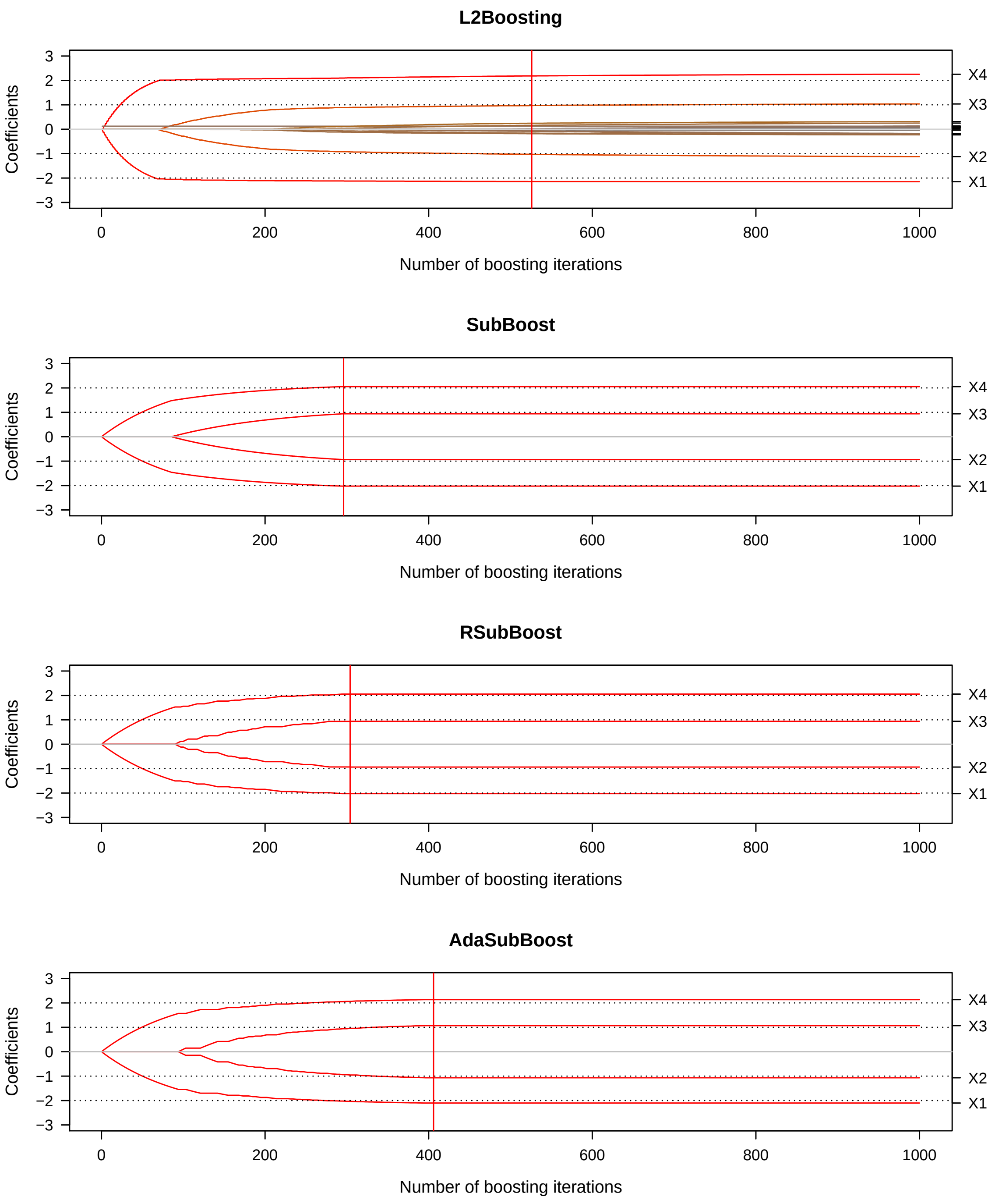

Figure S1: Low-dimensional illustrative data example. Coefficient paths $\beta_j^{[t]}$ for $j \in \mathcal{P}$ along the number of iterations t of L_2Boosting, SubBoost, RSubBoost and AdaSubBoost. Horizontal black dotted lines indicate the component values of the true coefficient vector $\boldsymbol{\beta}$. For L_2Boosting, the vertical red line indicates the CV-optimal stopping iteration m_{CV}; for SubBoost it indicates the automatic stopping iteration, while for RSubBoost and AdaSubBoost the automatic stopping after the first $N_{\mathrm{stop}} = p/2 = 10$ succeeding iterations without any updates is indicated.

2 Additional results for simulation study

2.1 Sparse high-dimensional setting (b)

For the sparse high-dimensional setting (b), Figure S2 shows that the two randomized subspace boosting algorithms again substantially reduce the number of false positives in comparison to L_2Boosting, but at the cost of an increase in false negatives (particularly for RSubBoost). Thus, in contrast to the sparse high-dimensional setting (a) with high correlations among signal variables, there is a decrease (instead of an increase) in statistical power for the subspace boosting algorithms in setting (b) with high correlations predominantly between signal and noise variables. In comparison to classical L_2Boosting, the earlier stopping approach via XGBoost, twin boosting and stability selection again lead to a reduction of false positives. Here, XGBoost and twin boosting tend to select more false positives and slightly less false negatives than the subspace boosting algorithms, while stability selection yields very small numbers of false positives in conjunction with a substantial increase in false negatives. The predictive performance of RSubBoost and AdaSubBoost is similar in setting (b) with slight advantages for AdaSubBoost, outperforming classical L_2Boosting and twin boosting, while the predictive ability of the very sparse models from stability selection is negatively affected due to larger numbers of false negatives. The subspace boosting algorithms again show a favorable variable selection and prediction performance compared to the lasso and the elastic net. While AdaSubBoost tends to yield smaller numbers of false positives and false negatives compared to the relaxed lasso, the predictive performance of the relaxed lasso is favorable in high-dimensional setting (b).

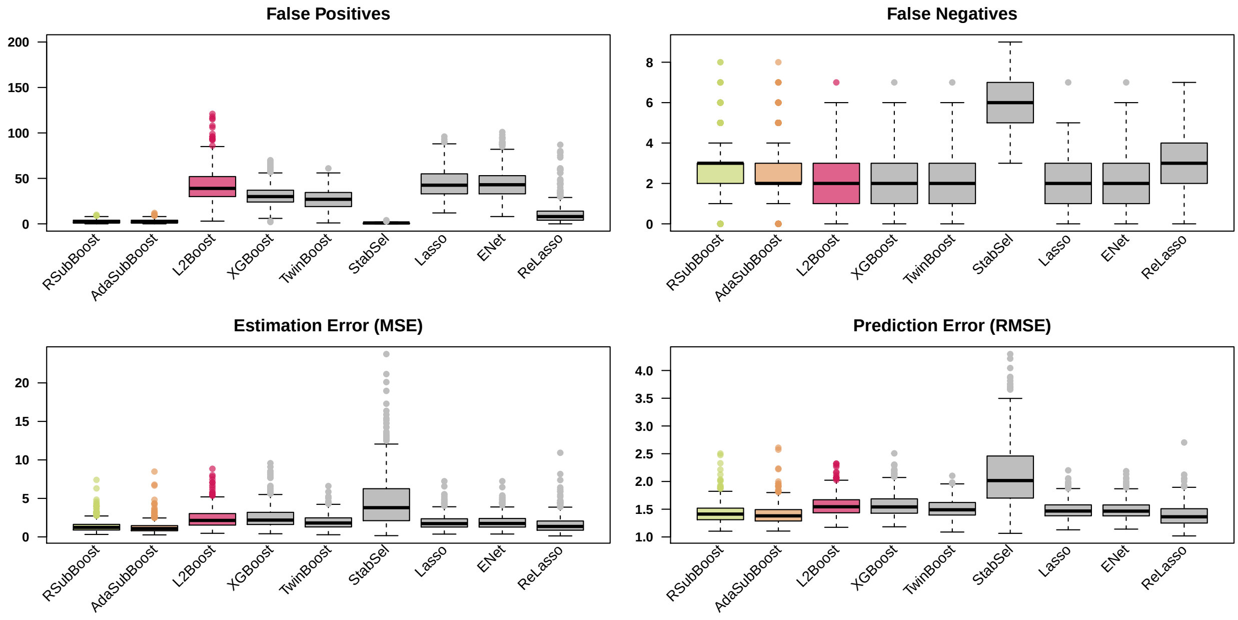

Figure S2: Results for high-dimensional simulation setting (b). Boxplots of false positives, false negatives, estimation error and prediction error on independent test set (of size 1000), for 500 simulation replicates with $n = 100$, $p = 1000$, random $S_{\text{true}} \subset \mathcal{P}$ with $|S_{\text{true}}| = 10$ and Toeplitz correlation with $\rho = 0.8$.

2.2 Detailed numerical results

	SubBoost	RSubBoost	AdaSubBoost	L_2Boosting	XGBoost	TwinBoost	StabSel	Lasso	ENet	ReLasso
Median FP (IQR)	2.000 (3.000)	3.000 (3.000)	3.000 (3.000)	6.000 (5.000)	4.000 (4.000)	2.000 (4.000)	**0.000** (0.000)	5.000 (5.000)	5.000 (5.000)	2.000 (4.000)
Mean FP (SD)	2.706 (2.000)	2.734 (1.998)	2.688 (1.973)	5.950 (3.548)	4.048 (2.613)	2.332 (2.430)	**0.108** (0.336)	5.514 (3.404)	5.696 (3.595)	2.624 (2.971)
P-value	<0.001	<0.001	<0.001	<0.001	<0.001	<0.001	**best**	<0.001	<0.001	<0.001
Median FN (IQR)	**0.000** (1.000)	**0.000** (1.000)	**0.000** (1.000)	**0.000** (1.000)	**0.000** (1.000)	1.000 (1.000)	1.000 (1.000)	**0.000** (1.000)	**0.000** (1.000)	1.000 (1.000)
Mean FN (SD)	**0.402** (0.617)	**0.402** (0.617)	0.410 (0.638)	0.460 (0.664)	0.672 (0.799)	0.800 (0.835)	1.200 (0.859)	0.480 (0.656)	0.454 (0.646)	0.738 (0.814)
P-value	**best**	1.000	0.362	0.005	<0.001	<0.001	<0.001	<0.001	0.016	<0.001
Median MSE (IQR)	0.281 (0.311)	0.276 (0.299)	**0.266** (0.291)	0.333 (0.368)	0.368 (0.452)	0.334 (0.448)	0.289 (0.496)	0.347 (0.354)	0.344 (0.362)	0.305 (0.374)
Mean MSE (SD)	0.354 (0.286)	0.345 (0.276)	**0.334** (0.268)	0.426 (0.338)	0.546 (0.598)	0.510 (0.601)	0.495 (0.586)	0.441 (0.351)	0.442 (0.350)	0.388 (0.355)
P-value	<0.001	<0.001	**best**	<0.001	<0.001	<0.001	<0.001	<0.001	<0.001	0.002
Median RMSE (IQR)	1.052 (0.048)	1.051 (0.047)	1.049 (0.049)	1.056 (0.050)	1.062 (0.057)	1.055 (0.058)	**1.048** (0.060)	1.060 (0.052)	1.060 (0.052)	1.055 (0.055)
Mean RMSE (SD)	1.054 (0.040)	1.053 (0.039)	**1.052** (0.039)	1.058 (0.040)	1.069 (0.053)	1.067 (0.069)	1.057 (0.057)	1.063 (0.041)	1.064 (0.042)	1.059 (0.042)
P-value	<0.001	<0.001	**best**	<0.001	<0.001	<0.001	0.956	<0.001	<0.001	<0.001
Mean Time in s (SD)	0.947 (0.716)	1.019 (0.626)	1.085 (0.560)	0.731 (0.078)	0.358 (0.186)	12.034 (0.694)	11.567 (0.928)	**0.147** (0.040)	0.852 (0.165)	0.882 (0.184)

Table S1: Detailed numerical results for low-dimensional simulation setting. Bold values indicate the best performing methods. P-values refer to two-sided Wilcoxon tests for paired samples, comparing each method with the best performing method which minimizes the mean of the respective evaluation metric. FP: False positives. FN: False negatives. MSE: Mean squared estimation error. RMSE: Root mean squared error of prediction. Time: Computational time (in seconds). IQR: Interquartile range. SD: standard deviation.

	RSubBoost	AdaSubBoost	L_2Boosting	XGBoost	TwinBoost	StabSel	Lasso	ENet	ReLasso
Median FP (IQR)	**0.000** (0.000)	**0.000** (0.250)	17.500 (23.000)	11.000 (13.000)	14.000 (18.000)	**0.000** (0.000)	23.000 (29.000)	24.000 (29.250)	1.000 (6.000)
Mean FP (SD)	0.268 (0.566)	0.328 (0.640)	22.740 (20.394)	13.104 (10.042)	16.598 (13.673)	**0.100** (0.326)	27.288 (20.738)	33.624 (62.689)	7.782 (15.940)
P-value	<0.001	<0.001	<0.001	<0.001	<0.001	**best**	<0.001	<0.001	<0.001
Median FN (IQR)	4.000 (3.000)	**3.000** (3.000)	5.000 (3.000)	5.000 (2.000)	5.000 (2.000)	6.000 (2.000)	5.000 (3.000)	5.000 (3.000)	5.000 (3.000)
Mean FN (SD)	3.784 (1.877)	**3.748** (1.926)	4.692 (1.751)	4.918 (1.814)	4.934 (1.763)	6.118 (1.715)	4.608 (1.726)	4.520 (1.684)	5.248 (1.805)
P-value	0.367	**best**	<0.001	<0.001	<0.001	<0.001	<0.001	<0.001	<0.001
Median MSE (IQR)	2.952 (3.025)	**2.697** (2.742)	5.569 (4.621)	5.924 (4.928)	5.436 (4.762)	6.241 (5.082)	5.420 (4.580)	5.421 (4.752)	5.221 (5.106)
Mean MSE (SD)	3.851 (2.762)	**3.633** (2.817)	6.286 (3.338)	6.521 (3.510)	6.144 (3.486)	6.730 (3.627)	6.005 (3.381)	6.002 (3.397)	5.813 (3.460)
P-value	<0.001	**best**	<0.001	<0.001	<0.001	<0.001	<0.001	<0.001	<0.001
Median RMSE (IQR)	1.316 (0.222)	**1.292** (0.214)	1.598 (0.332)	1.567 (0.354)	1.641 (0.358)	1.543 (0.432)	1.555 (0.341)	1.560 (0.354)	1.488 (0.387)
Mean RMSE (SD)	1.355 (0.189)	**1.339** (0.194)	1.630 (0.243)	1.610 (0.261)	1.685 (0.279)	1.588 (0.303)	1.588 (0.247)	1.591 (0.253)	1.530 (0.272)
P-value	<0.001	**best**	<0.001	<0.001	<0.001	<0.001	<0.001	<0.001	<0.001
Mean Time in s (SD)	8.175 (6.849)	10.292 (5.194)	18.513 (2.966)	1.082 (0.554)	133.713 (24.523)	37.568 (6.290)	**0.399** (0.101)	5.076 (0.877)	4.034 (1.013)

Table S2: Detailed numerical results for sparse high-dimensional simulation setting (a). Bold values indicate the best performing methods. P-values refer to two-sided Wilcoxon tests for paired samples, comparing each method with the best performing method which minimizes the mean of the respective evaluation metric. FP: False positives. FN: False negatives. MSE: Mean squared estimation error. RMSE: Root mean squared error of prediction. Time: Computational time (in seconds). IQR: Interquartile range. SD: standard deviation.

	RSubBoost	AdaSubBoost	L_2Boosting	XGBoost	TwinBoost	StabSel	Lasso	ENet	ReLasso
Median FP (IQR)	2.000 (3.000)	2.000 (3.000)	39.000 (22.000)	30.000 (13.000)	27.000 (15.250)	**1.000** (1.000)	42.500 (22.000)	43.000 (20.000)	8.000 (10.000)
Mean FP (SD)	2.704 (1.867)	2.816 (1.943)	42.504 (17.917)	31.356 (11.328)	26.704 (11.375)	**0.706** (0.810)	45.114 (16.483)	45.144 (17.105)	11.942 (13.305)
P-value	<0.001	<0.001	<0.001	<0.001	<0.001	**best**	<0.001	<0.001	<0.001
Median FN (IQR)	3.000 (1.000)	**2.000** (1.000)	**2.000** (2.000)	**2.000** (2.000)	**2.000** (2.000)	6.000 (2.000)	**2.000** (2.000)	**2.000** (2.000)	3.000 (2.000)
Mean FN (SD)	2.602 (1.517)	2.582 (1.493)	1.926 (1.210)	2.102 (1.224)	2.132 (1.212)	6.060 (1.152)	1.842 (1.163)	**1.836** (1.173)	2.850 (1.490)
P-value	<0.001	<0.001	<0.001	<0.001	<0.001	<0.001	0.581	**best**	<0.001
Median MSE (IQR)	1.235 (0.731)	**1.076** (0.667)	2.138 (1.495)	2.181 (1.573)	1.804 (1.175)	3.795 (4.135)	1.723 (1.070)	1.741 (1.106)	1.356 (1.190)
Mean MSE (SD)	1.382 (0.771)	**1.256** (0.797)	2.414 (1.230)	2.586 (1.440)	1.947 (0.923)	4.705 (3.577)	1.947 (0.944)	1.950 (0.950)	1.635 (1.186)
P-value	<0.001	**best**	<0.001	<0.001	<0.001	<0.001	<0.001	<0.001	<0.001
Median RMSE (IQR)	1.412 (0.208)	1.380 (0.205)	1.544 (0.235)	1.541 (0.258)	1.490 (0.226)	2.016 (0.756)	1.467 (0.196)	1.465 (0.197)	**1.363** (0.258)
Mean RMSE (SD)	1.437 (0.182)	1.409 (0.182)	1.569 (0.193)	1.576 (0.211)	1.510 (0.174)	2.121 (0.592)	1.494 (0.162)	1.492 (0.165)	**1.394** (0.201)
P-value	<0.001	0.001	<0.001	<0.001	<0.001	<0.001	<0.001	<0.001	**best**
Mean Time in s (SD)	5.087 (5.863)	9.698 (8.427)	14.245 (9.929)	2.143 (3.232)	92.321 (33.320)	32.280 (19.242)	**0.297** (0.127)	3.925 (1.138)	2.887 (1.888)

Table S3: Detailed numerical results for sparse high-dimensional simulation setting (b). Bold values indicate the best performing methods. P-values refer to two-sided Wilcoxon tests for paired samples, comparing each method with the best performing method which minimizes the mean of the respective evaluation metric. FP: False positives. FN: False negatives. MSE: Mean squared estimation error. RMSE: Root mean squared error of prediction. Time: Computational time (in seconds). IQR: Interquartile range. SD: standard deviation.

	RSubBoost AIC	AdaSubBoost AIC	RSubBoost EBIC_1	AdaSubBoost EBIC_1	L_2Boosting	XGBoost	TwinBoost	StabSel	Lasso	ENet	ReLasso
Median FP (IQR)	38.000 (14.250)	13.000 (11.000)	**0.000** (0.000)	**0.000** (0.000)	286.000 (25.000)	189.000 (35.000)	**0.000** (1.000)	**0.000** (0.000)	104.500 (33.000)	105.000 (33.000)	95.000 (28.000)
Mean FP (SD)	38.212 (10.960)	13.726 (7.598)	0.250 (0.533)	0.170 (0.412)	286.138 (16.958)	189.680 (26.765)	0.578 (0.823)	**0.002** (0.045)	105.994 (25.480)	105.914 (25.446)	94.974 (21.475)
P-value	<0.001	<0.001	<0.001	<0.001	<0.001	<0.001	<0.001	**best**	<0.001	<0.001	<0.001
Median FN (IQR)	6.000 (4.000)	6.000 (4.000)	45.000 (9.000)	44.000 (7.000)	**5.000** (3.000)	8.000 (4.000)	17.000 (4.000)	61.000 (6.000)	13.000 (5.000)	13.000 (6.000)	14.000 (6.000)
Mean FN (SD)	6.320 (3.134)	6.340 (3.373)	45.084 (6.283)	43.556 (5.843)	**5.028** (2.115)	7.792 (2.936)	16.724 (3.269)	61.164 (4.420)	13.044 (3.809)	13.062 (3.817)	13.992 (3.855)
P-value	<0.001	<0.001	<0.001	<0.001	**best**	<0.001	<0.001	<0.001	<0.001	<0.001	<0.001
Median MSE (IQR)	13.193 (6.808)	5.410 (3.313)	69.206 (16.287)	65.279 (16.423)	4.293 (1.265)	6.356 (1.963)	3.801 (1.336)	53.188 (17.022)	8.899 (4.036)	8.882 (3.947)	**2.406** (1.135)
Mean MSE (SD)	14.480 (5.478)	6.999 (4.820)	69.249 (11.448)	65.659 (11.550)	4.398 (0.941)	6.548 (1.553)	3.970 (1.402)	53.813 (12.104)	9.627 (3.296)	9.633 (3.287)	**2.668** (1.183)
P-value	<0.001	<0.001	<0.001	<0.001	<0.001	<0.001	<0.001	<0.001	<0.001	<0.001	**best**
Median RMSE (IQR)	1.785 (0.290)	1.377 (0.201)	3.852 (0.478)	3.740 (0.496)	1.426 (0.103)	1.499 (0.117)	1.304 (0.113)	3.260 (0.503)	1.612 (0.201)	1.613 (0.200)	**1.278** (0.087)
Mean RMSE (SD)	1.832 (0.227)	1.450 (0.233)	3.855 (0.348)	3.748 (0.362)	1.438 (0.087)	1.502 (0.087)	1.321 (0.109)	3.279 (0.370)	1.636 (0.160)	1.637 (0.159)	**1.290** (0.078)
P-value	<0.001	<0.001	<0.001	<0.001	<0.001	<0.001	<0.001	<0.001	<0.001	<0.001	**best**
Mean Time in s (SD)	155.769 (18.125)	159.319 (11.322)	159.588 (33.341)	202.147 (35.683)	310.442 (32.498)	475.383 (83.008)	1062.141 (65.635)	283.587 (27.016)	**3.347** (0.386)	41.629 (4.493)	19.089 (3.215)

Table S4: Detailed numerical results for non-sparse high-dimensional simulation setting. Bold values indicate the best performing methods. P-values refer to two-sided Wilcoxon tests for paired samples, comparing each method with the best performing method which minimizes the mean of the respective evaluation metric. FP: False positives. FN: False negatives. MSE: Mean squared estimation error. RMSE: Root mean squared error of prediction. Time: Computational time (in seconds). IQR: Interquartile range. SD: standard deviation.

2.3 Computation times

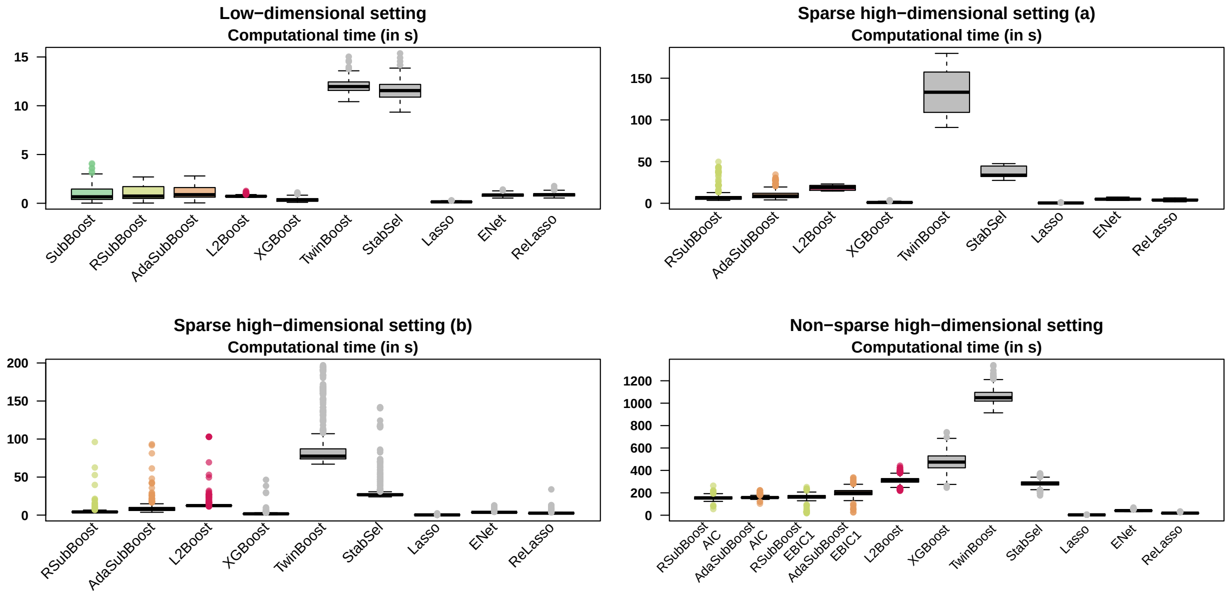

Figure S3: Computational times (in seconds) for the application of the different methods on simulated datasets under the four considered simulation settings (see main document for details).

2.4 Model sizes of subspace boosting algorithms

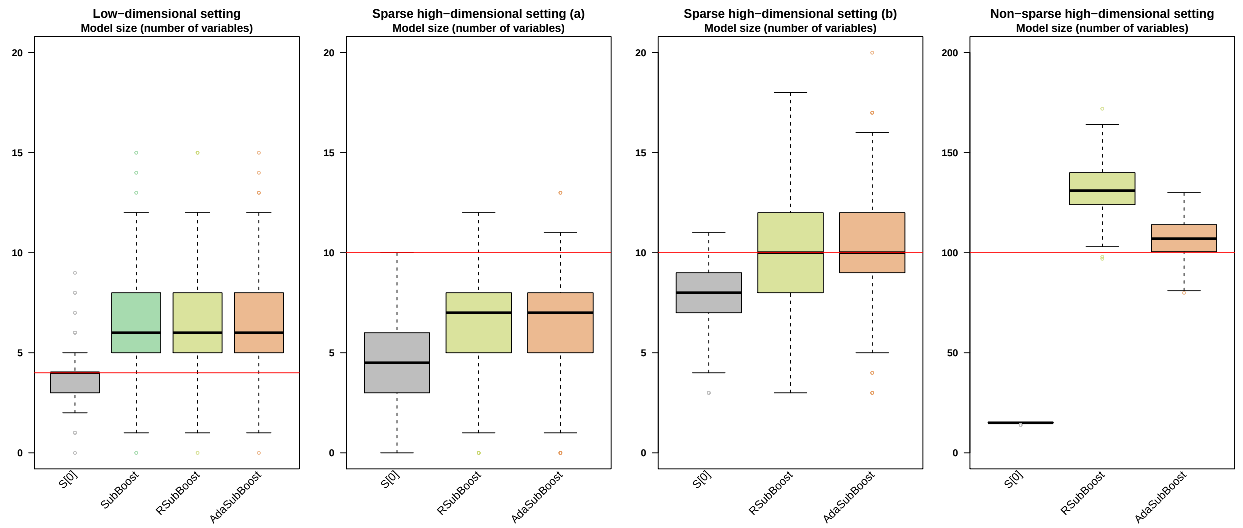

Figure S4: Model sizes (number of selected variables) for the "baseline" model $S^{[0]}$ (starting model for RSubBoost and AdaSubBoost) as well as for final RSubBoost and AdaSubBoost models under the four considered simulation settings (see main document for details). Results are based on the BIC for the low-dimensional setting, the EBIC_1 for the sparse high-dimensional settings (a) and (b) and the AIC for the non-sparse high-dimensional setting. Red horizontal lines indicate the model sizes of the true data generating models S_{true}.

2.5 Influence of tuning parameters in subspace boosting algorithms

2.5.1 Influence of selection criterion

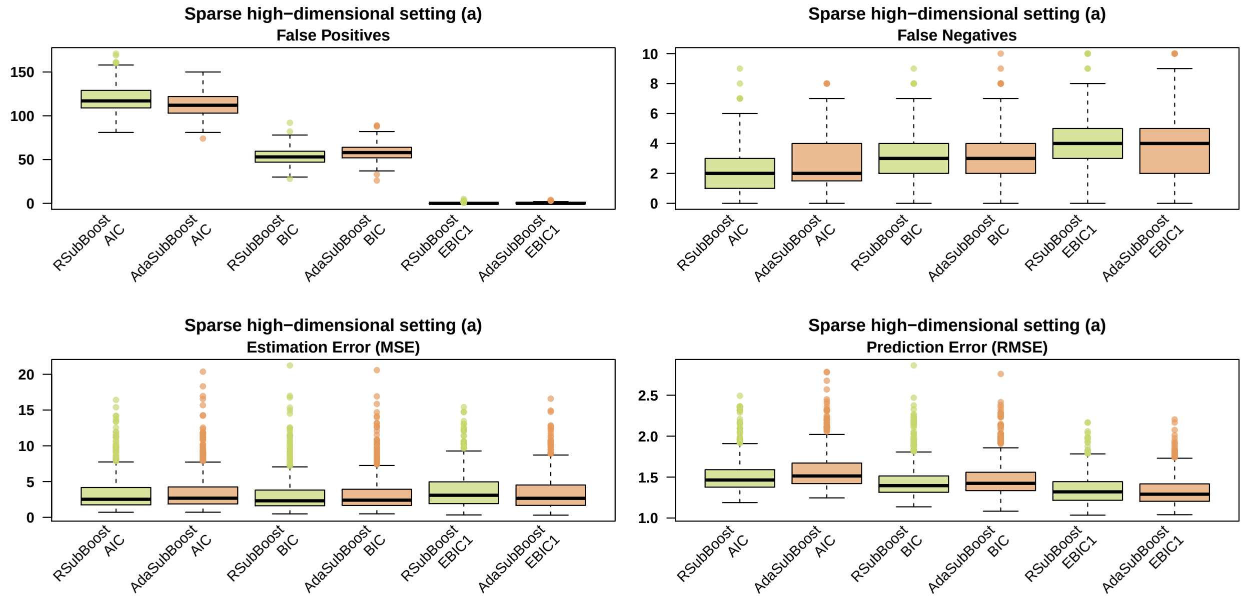

Figure S5: Influence of selection criterion for sparse high-dimensional simulation setting (a). Boxplots of false positives, false negatives, estimation error and prediction error on independent test set (of size 1000), for RSubBoost and AdaSubBoost in combination with AIC, BIC and EBIC_1. All remaining tuning parameters of the algorithms are the same as in the simulation study of the main document, considering 500 simulation replicates with $n = 100$, $p = 1000$, $S_{\text{true}} = \{1, \ldots, 10\}$ and Toeplitz correlation with $\rho = 0.8$.

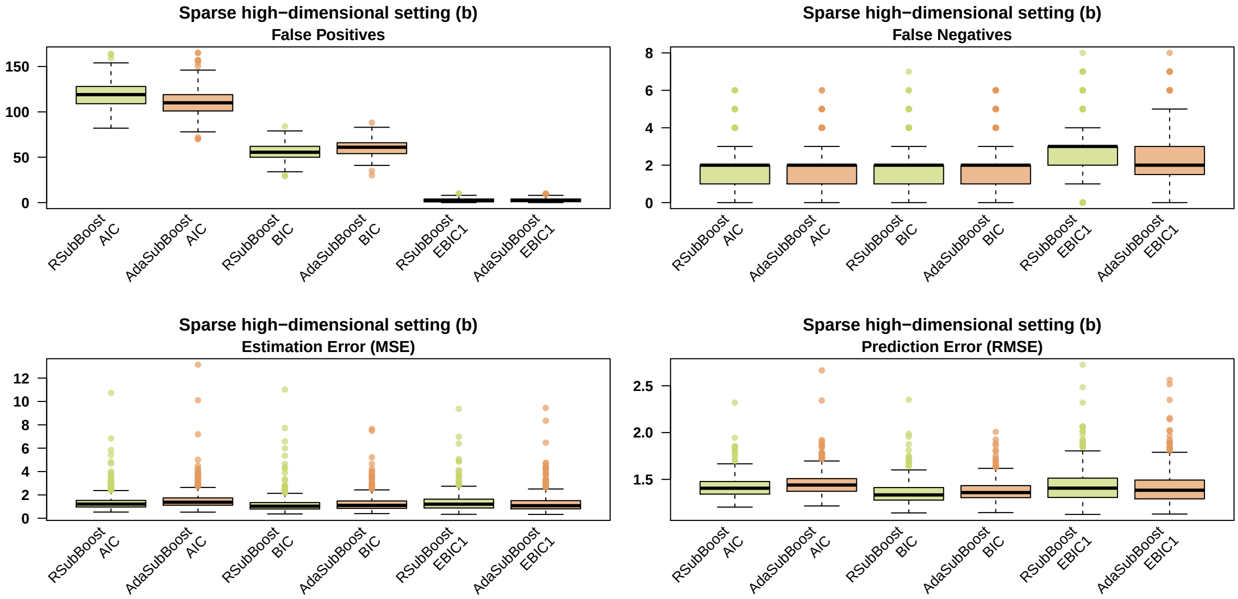

Figure S6: Influence of selection criterion for sparse high-dimensional simulation setting (b). Boxplots of false positives, false negatives, estimation error and prediction error on independent test set (of size 1000), for RSubBoost and AdaSubBoost in combination with AIC, BIC and EBIC_1. All remaining tuning parameters of the algorithms are the same as in the simulation study of the main document, considering 500 simulation replicates with $n = 100$, $p = 1000$, random $S_{\text{true}} \subset \mathcal{P}$ with $|S_{\text{true}}| = 10$ and Toeplitz correlation with $\rho = 0.8$.

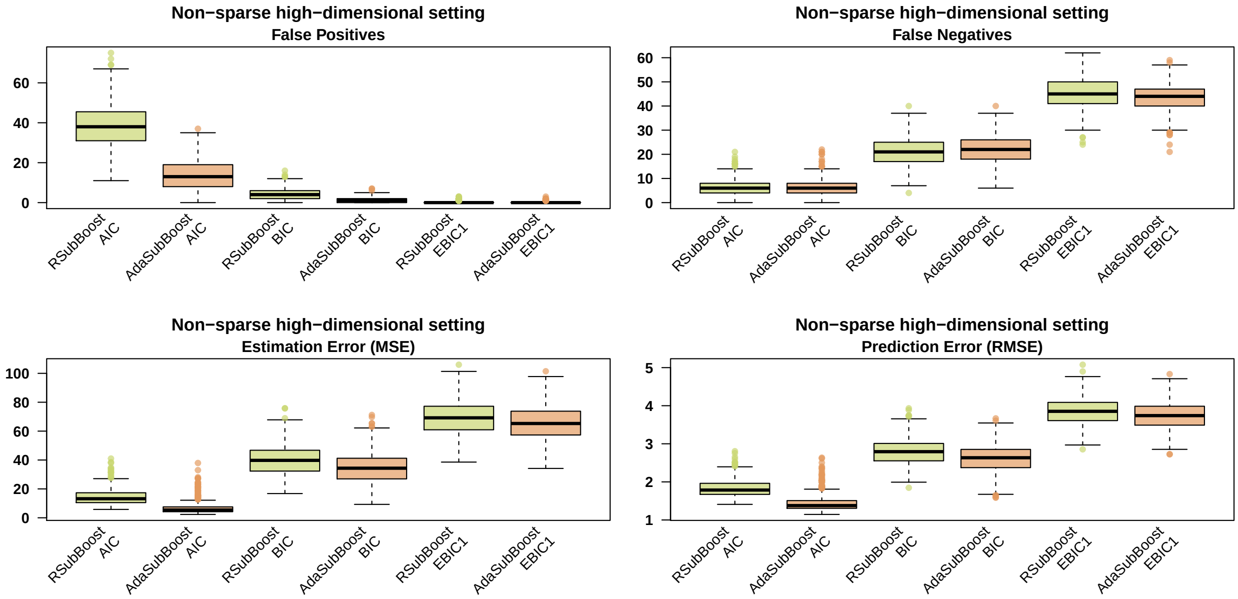

Figure S7: Influence of selection criterion for non-sparse high-dimensional simulation setting. Boxplots of false positives, false negatives, estimation error and prediction error on independent test set (of size 1000), for RSubBoost and AdaSubBoost in combination with AIC, BIC and EBIC_1. All remaining tuning parameters of the algorithms are the same as in the simulation study of the main document, considering 500 simulation replicates with $n = 1000$, $p = 1000$, $S_{\text{true}} = \{1, \ldots, 100\}$ and Toeplitz correlation with $\rho = 0.8$.

2.5.2 Influence of adaptation parameter K

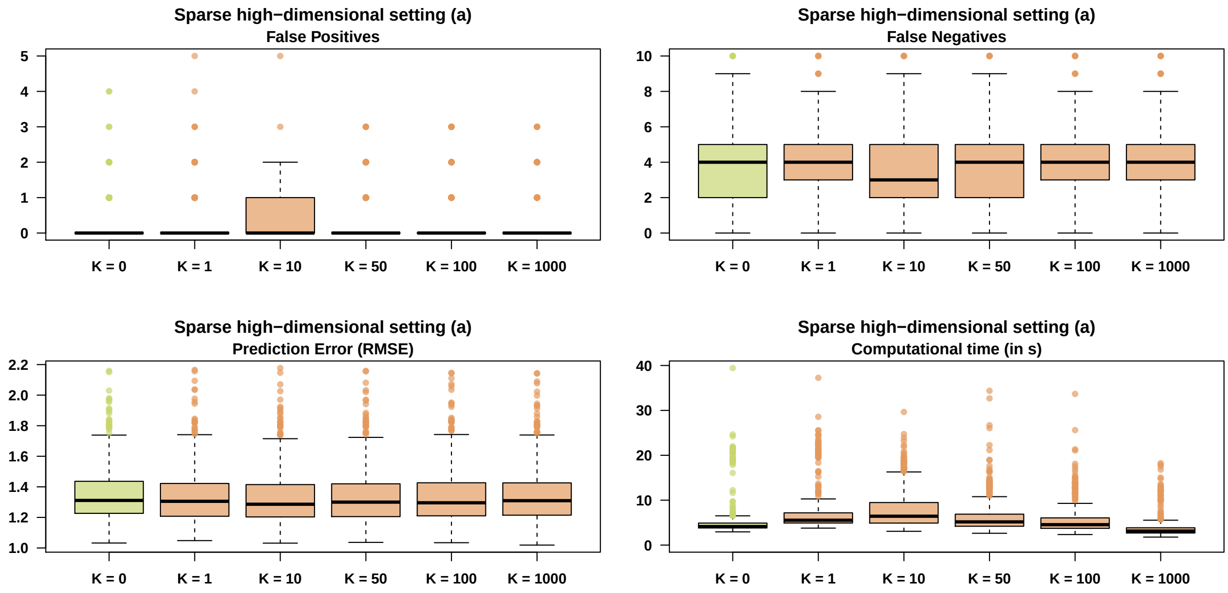

Figure S8: Influence of adaptation parameter K in AdaSubBoost with the EBIC_1 for sparse high-dimensional simulation setting (a). Boxplots of false positives, false negatives, prediction error on independent test set (of size 1000) and computational time (in seconds) for AdaSubBoost with varying adaptation parameter $K \in \{0, 1, 10, 50, 100, 1000\}$. Note that $K = 0$ corresponds to RSubBoost. All remaining tuning parameters of AdaSubBoost are the same as in the simulation study of the main document ($q = 20$, $s_{\max} = 15$), considering 500 simulation replicates with $n = 100$, $p = 1000$, $S_{\text{true}} = \{1, \dots, 10\}$ and Toeplitz correlation with $\rho = 0.8$.

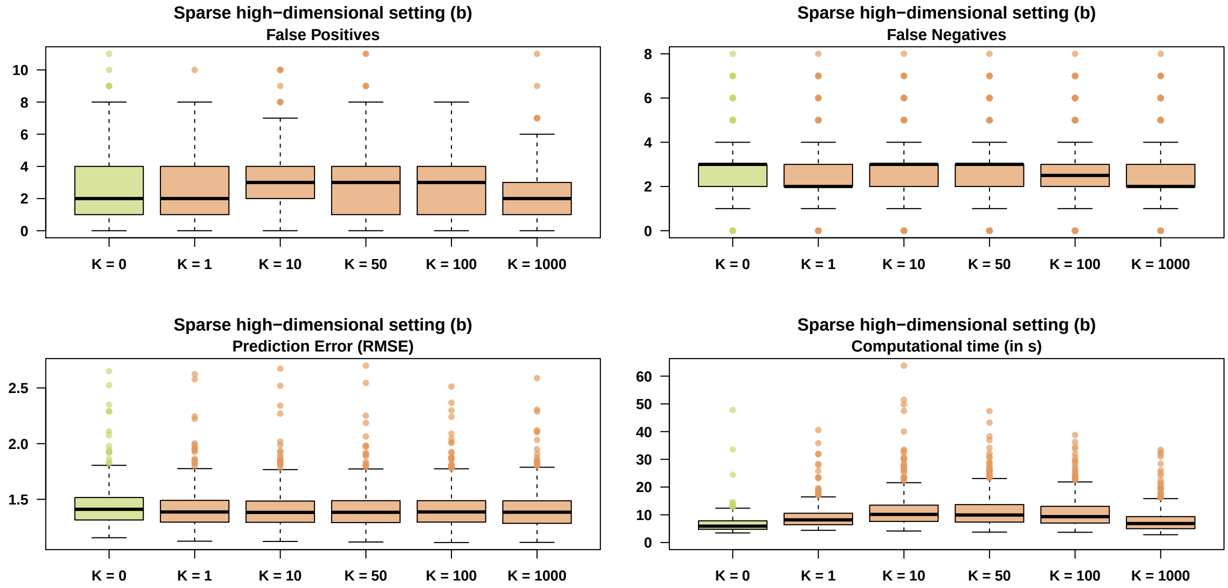

Figure S9: Influence of adaptation parameter K in AdaSubBoost with the EBIC_1 for sparse high-dimensional simulation setting (b). Boxplots of false positives, false negatives, prediction error on independent test set (of size 1000) and computational time (in seconds) for AdaSubBoost with varying adaptation parameter $K \in \{0, 1, 10, 50, 100, 1000\}$. Note that $K = 0$ corresponds to RSubBoost. All remaining tuning parameters of AdaSubBoost are the same as in the simulation study of the main document ($q = 20$, $s_{\max} = 15$), considering 500 simulation replicates with $n = 100$, $p = 1000$, random $S_{\text{true}} \subset \mathcal{P}$ with $|S_{\text{true}}| = 10$ and Toeplitz correlation with $\rho = 0.8$.

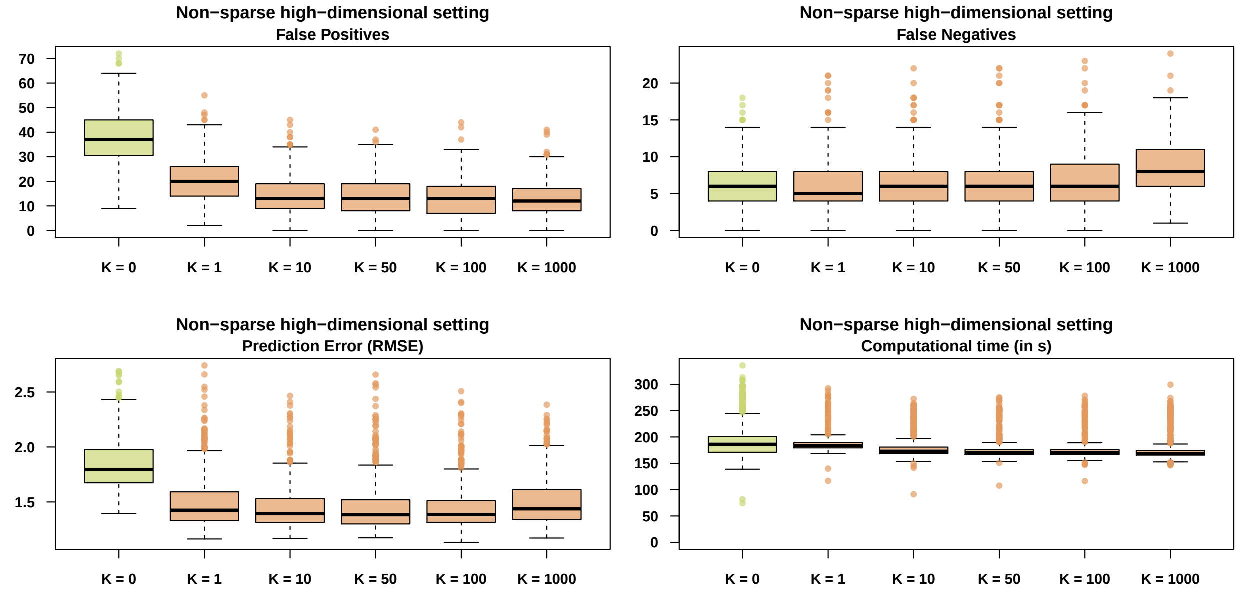

Figure S10: Influence of adaptation parameter K in AdaSubBoost with the AIC for non-sparse high-dimensional simulation setting. Boxplots of false positives, false negatives, prediction error on independent test set (of size 1000) and computational time (in seconds) for AdaSubBoost with varying adaptation parameter $K \in \{0, 1, 10, 50, 100, 1000\}$. Note that $K = 0$ corresponds to RSubBoost. All remaining tuning parameters of AdaSubBoost are the same as in the simulation study of the main document ($q = 20$, $s_{\max} = 15$), considering 500 simulation replicates with $n = 1000$, $p = 1000$, $S_{\text{true}} = \{1, \dots, 100\}$ with $|S_{\text{true}}| = 100$ and Toeplitz correlation with $\rho = 0.8$.

2.5.3 Influence of initial search size q and maximum update size $s_{\max}$

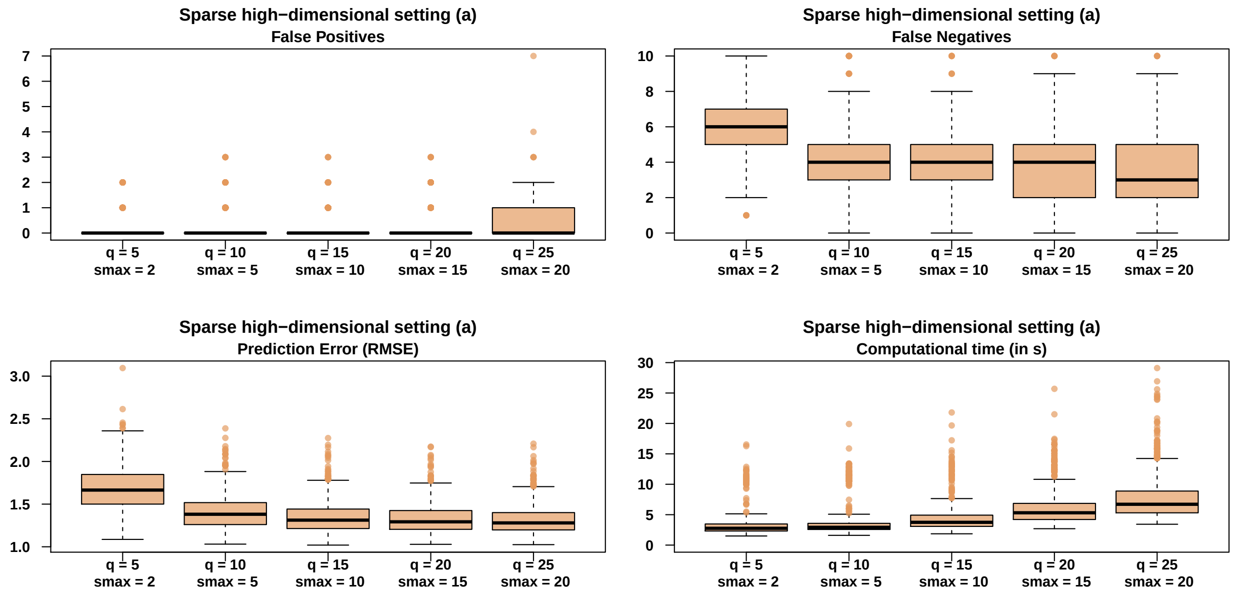

Figure S11: Influence of initial search size q and maximum update size $s_{\max}$ in AdaSubBoost with the EBIC$_1$ for sparse high-dimensional simulation setting (a). Boxplots of false positives, false negatives, prediction error on independent test set (of size 1000) and computational time (in seconds) for AdaSubBoost with varying initial search size $q \in \{5, 10, 15, 20, 25\}$ and maximum update size $s_{\max} \in \{2, 5, 10, 15, 20\}$. All remaining tuning parameters of AdaSubBoost are the same as in the simulation study of the main document ($K = p/q$), considering 500 simulation replicates with $n = 100$, $p = 1000$, $S_{\text{true}} = \{1, \ldots, 10\}$ and Toeplitz correlation with $\rho = 0.8$.

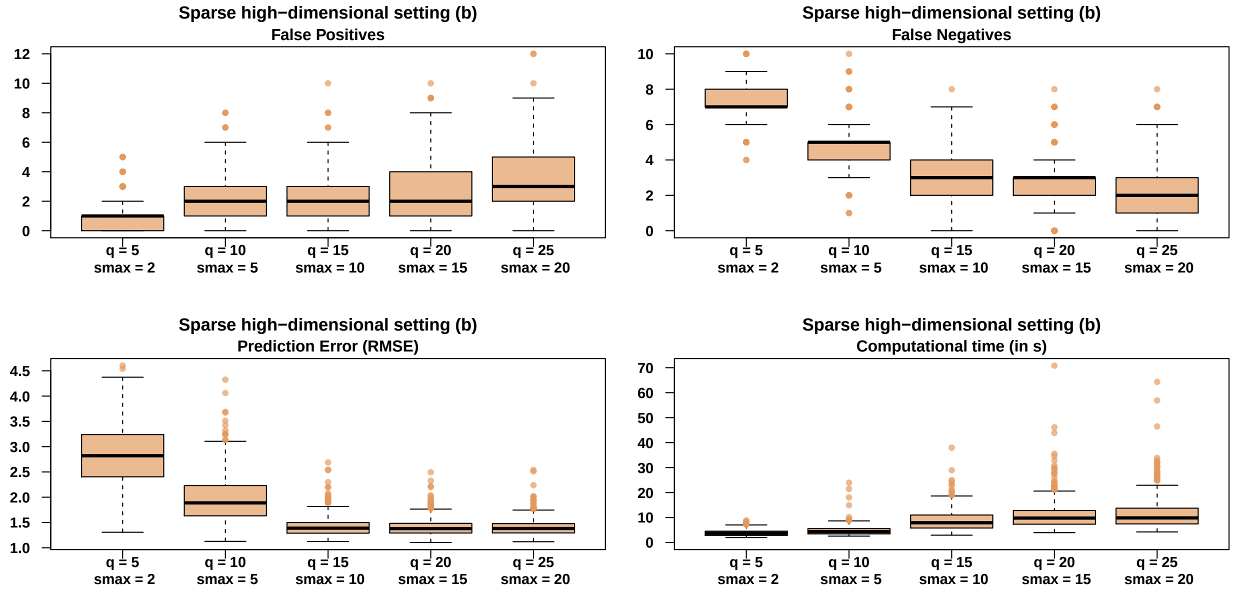

Figure S12: Influence of initial search size q and maximum update size $s_{\max}$ in AdaSubBoost with the EBIC$_1$ for sparse high-dimensional simulation setting (b). Boxplots of false positives, false negatives, prediction error on independent test set (of size 1000) and computational time (in seconds) for AdaSubBoost with varying initial search size $q \in \{5, 10, 15, 20, 25\}$ and maximum update size $s_{\max} \in \{2, 5, 10, 15, 20\}$. All remaining tuning parameters of AdaSubBoost are the same as in the simulation study of the main document ($K = p/q$), considering 500 simulation replicates with $n = 100$, $p = 1000$, random $S_{\text{true}} \subset \mathcal{P}$ with $|S_{\text{true}}| = 10$ and Toeplitz correlation with $\rho = 0.8$.

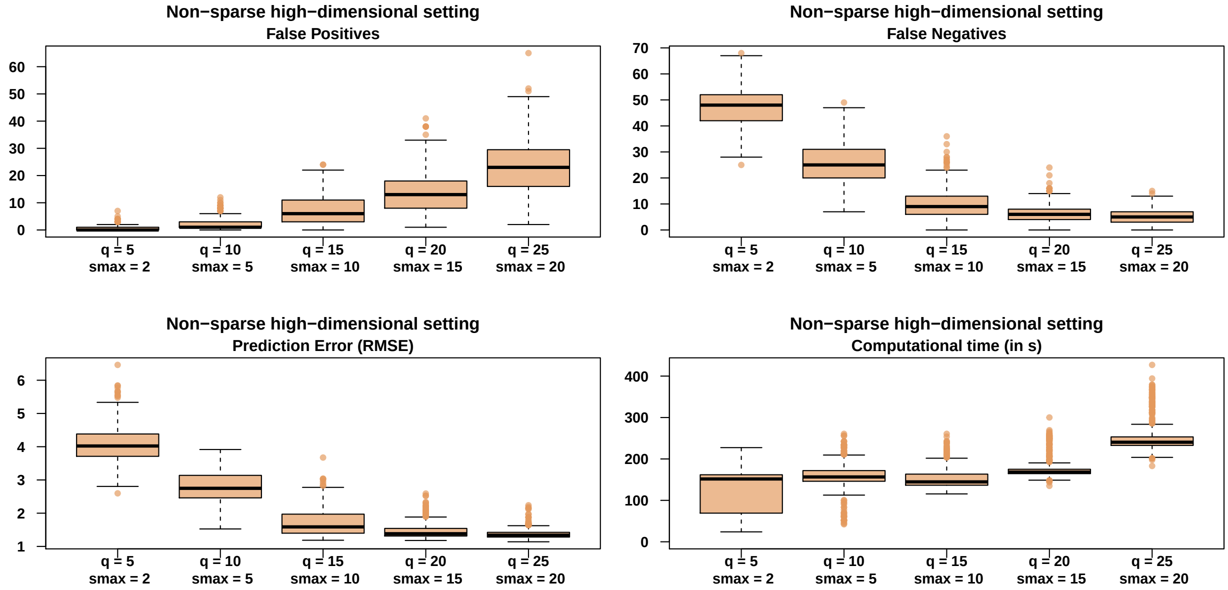

Figure S13: Influence of initial search size q and maximum update size $s_{\max}$ in AdaSubBoost with the AIC for non-sparse high-dimensional simulation setting. Boxplots of false positives, false negatives, prediction error on independent test set (of size 1000) and computational time (in seconds) for AdaSubBoost with varying initial search size $q \in \{5, 10, 15, 20, 25\}$ and maximum update size $s_{\max} \in \{2, 5, 10, 15, 20\}$. All remaining tuning parameters of AdaSubBoost are the same as in the simulation study of the main document ($K = p/q$), considering 500 simulation replicates with $n = 1000$, $p = 1000$, $S_{\text{true}} = \{1, \ldots, 100\}$ with $|S_{\text{true}}| = 100$ and Toeplitz correlation with $\rho = 0.8$.

3 Additional results for biomedical data applications

3.1 Detailed numerical results

	SubBoost	RSubBoost	AdaSubBoost	L_2Boosting	XGBoost	TwinBoost	StabSel	Lasso	ENet	ReLasso
**Bodyfat data**										
Median model size (IQR)	6.00 (0.50)	6.00 (1.00)	6.00 (0.00)	7.00 (0.00)	**2.00** (0.00)	**2.00** (1.00)	**2.00** (0.00)	7.00 (1.00)	8.00 (0.00)	6.00 (1.00)
Mean model size (SD)	6.25 (0.67)	6.37 (0.64)	6.06 (0.65)	7.14 (0.74)	2.00 (0.00)	**1.94** (0.84)	2.13 (0.34)	6.63 (0.62)	8.03 (0.61)	5.62 (0.59)
P-value	<0.001	<0.001	<0.001	<0.001	0.616	**best**	0.121	<0.001	<0.001	<0.001
Median LOOCV error (IQR)	2.17 (2.85)	2.21 (2.79)	2.28 (2.82)	**1.96** (2.94)	4.97 (3.63)	9.13 (8.65)	2.49 (3.49)	2.19 (3.02)	2.02 (2.91)	2.04 (2.87)
Mean LOOCV error (SD)	**2.54** (2.41)	**2.54** (2.40)	2.57 (2.41)	2.60 (2.45)	5.51 (4.10)	9.20 (6.22)	3.14 (2.56)	2.57 (2.45)	2.60 (2.39)	2.57 (2.46)
P-value	0.349	**best**	0.063	0.168	<0.001	<0.001	0.002	0.239	0.23	0.523
Mean Time in s (SD)	1.20 (0.04)	1.28 (0.05)	1.47 (0.08)	0.60 (0.02)	1.47 (0.16)	11.76 (1.13)	9.81 (0.45)	**0.12** (0.03)	0.73 (0.09)	0.51 (0.07)
**Diabetes data**										
Median model size (IQR)	9.00 (0.00)	9.00 (0.00)	8.00 (0.00)	8.00 (1.00)	8.00 (1.00)	**4.00** (4.00)	**4.00** (0.00)	8.00 (2.00)	8.00 (2.00)	7.00 (0.00)
Mean model size (SD)	9.00 (0.00)	8.86 (0.36)	8.04 (0.64)	7.92 (0.70)	7.83 (0.69)	4.41 (2.20)	**4.00** (0.00)	7.96 (1.03)	8.12 (1.07)	7.30 (1.05)
P-value	<0.001	<0.001	<0.001	<0.001	<0.001	0.001	**best**	<0.001	<0.001	<0.001
Median LOOCV error (IQR)	40.35 (44.95)	39.58 (45.34)	39.92 (45.32)	40.40 (43.98)	40.74 (45.34)	42.65 (48.83)	39.72 (45.36)	40.09 (45.33)	40.32 (45.97)	**39.43** (45.36)
Mean LOOCV error (SD)	44.69 (32.31)	44.49 (32.05)	44.49 (32.03)	**44.45** (31.99)	44.48 (31.98)	48.13 (32.97)	45.04 (31.45)	44.60 (32.19)	44.61 (32.26)	44.47 (32.33)
P-value	0.188	0.90	0.865	**best**	0.40	<0.001	0.179	0.134	0.293	0.686
Mean Time in s (SD)	1.96 (0.34)	1.65 (0.16)	1.99 (0.17)	0.93 (0.08)	0.34 (0.05)	11.32 (0.57)	12.91 (0.73)	**0.10** (0.02)	0.72 (0.12)	0.52 (0.08)
**Riboflavin data**										
Median model size (IQR)	—	40.00 (9.00)	23.00 (8.00)	39.00 (6.50)	5.00 (2.00)	**4.00** (1.00)	**4.00** (1.50)	40.00 (4.00)	42.00 (141.00)	31.00 (5.00)
Mean model size (SD)	—	40.21 (7.54)	23.39 (6.48)	41.90 (11.98)	6.58 (4.31)	4.49 (0.65)	**4.04** (0.95)	40.59 (3.89)	101.27 (102.86)	31.55 (5.52)
P-value	—	<0.001	<0.001	<0.001	<0.001	0.003	**best**	<0.001	<0.001	<0.001
Median LOOCV error (IQR)	—	**0.23** (0.36)	0.28 (0.32)	**0.23** (0.34)	0.63 (0.60)	0.37 (0.36)	0.35 (0.29)	0.24 (0.36)	0.29 (0.40)	0.33 (0.34)
Mean LOOCV error (SD)	—	**0.32** (0.32)	0.35 (0.31)	**0.32** (0.32)	0.67 (0.50)	0.44 (0.40)	0.44 (0.41)	**0.32** (0.30)	0.35 (0.32)	0.36 (0.28)
P-value	—	0.906	0.20	0.825	<0.001	0.002	0.005	**best**	0.034	0.011
Mean Time in s (SD)	—	45.50 (10.28)	51.85 (13.49)	23.34 (0.56)	28.85 (26.77)	118.96 (16.67)	52.99 (2.23)	**0.49** (0.04)	7.13 (0.64)	3.91 (1.10)
**PCR data**										
Median model size (IQR)	—	1.00 (0.00)	1.00 (0.00)	44.00 (64.25)	11.00 (7.00)	32.00 (32.25)	**0.00** (0.00)	54.00 (39.50)	186.00 (237.25)	8.00 (46.25)
Mean model size (SD)	—	1.18 (0.47)	1.00 (0.18)	67.23 (82.07)	12.78 (7.06)	26.28 (16.77)	**0.02** (0.13)	43.45 (20.84)	235.12 (180.23)	20.82 (22.55)
P-value	—	<0.001	<0.001	<0.001	<0.001	<0.001	**best**	<0.001	<0.001	<0.001
Median LOOCV error (IQR)	—	0.42 (0.54)	**0.41** (0.57)	0.63 (0.66)	0.49 (0.61)	0.60 (0.59)	0.46 (0.70)	0.49 (0.65)	0.50 (0.67)	0.63 (0.76)
Mean LOOCV error (SD)	—	**0.55** (0.45)	0.56 (0.46)	0.61 (0.44)	0.57 (0.45)	0.61 (0.43)	0.62 (0.50)	0.56 (0.43)	0.59 (0.43)	0.67 (0.47)
P-value	—	**best**	0.691	0.482	0.817	0.298	0.013	0.749	0.573	0.014
Mean Time in s (SD)	—	186.86 (23.36)	190.45 (6.73)	113.91 (17.68)	3.41 (1.38)	404.90 (26.63)	312.38 (40.09)	**1.88** (0.28)	24.49 (2.05)	6.34 (0.56)

Table S5: Detailed numerical results of leave-one-out cross-validation (LOOCV) for biomedical data applications in terms of model sizes, absolute prediction errors and computational times. Bold values indicate the best performing methods. P-values refer to two-sided Wilcoxon tests for paired samples, comparing each method with the best performing method which minimizes the mean of the respective evaluation metric. IQR: Interquartile range. Time: Computational time (in seconds). SD: standard deviation.

3.2 Computation times

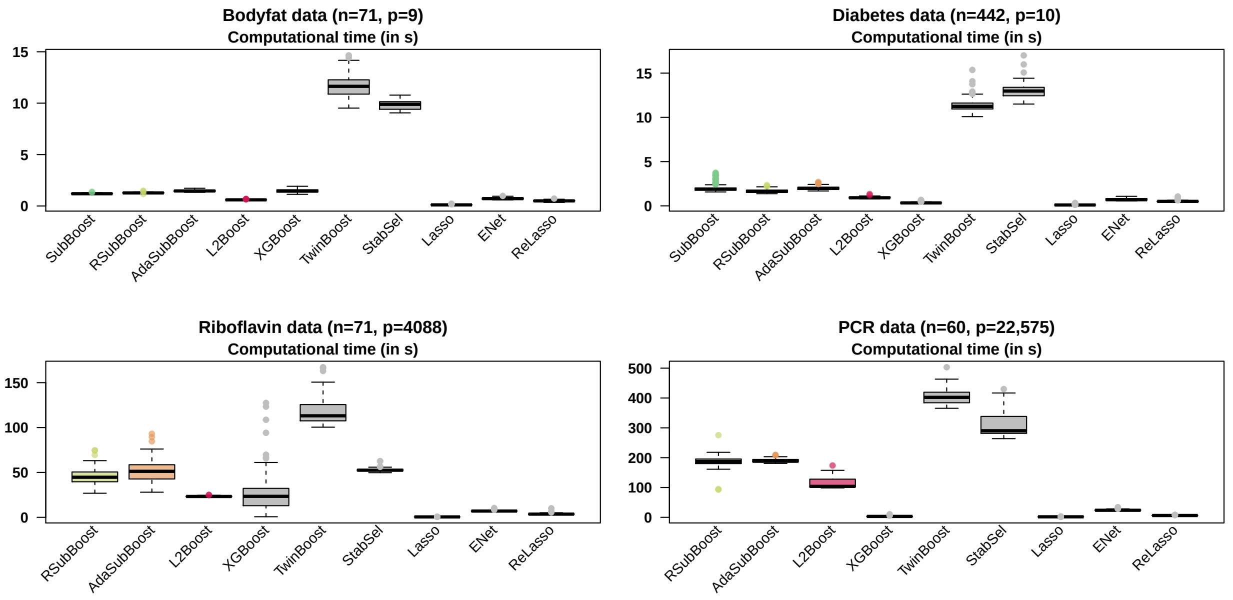

Figure S14: Computational times (in seconds) for the application of the different methods on leave-one-out cross-validation datasets for the four considered biomedical data applications (see main document for details).
